# Bcl6 drives stem-like memory macrophages differentiation to foster tumor progression

**DOI:** 10.1007/s00018-022-04660-0

**Published:** 2022-12-21

**Authors:** Weiwei Zhang, Qin Han, Yina Ding, Huihui Zhou, Zhipeng Chen, Jingjing Wang, Jiaxin Xiang, Zhengbo Song, Muhammad Abbas, Liyun Shi

**Affiliations:** 1 School of Medicine, Nanjing University of Chinese Medicine, Nanjing, 210023 Jiangsu China; 2 Institute of Cancer and Basic Medicine (IBMC), Chinese Academy of Sciences, The Cancer Hospital of the University of Chinese Academy of Sciences (Zhejiang Cancer Hospital), Hangzhou, 310022 Zhejiang China; 3 Key Lab of Inflammation and Immunoregulation, Hangzhou Normal University School of Medicine, Hangzhou, 310012 Zhejiang China; 4 College of Pharmacy, Nanjing University of Chinese Medicine, Nanjing, 210023 China; 5 Riphah Institute of Pharmaceutical Sciences, Riphah International University, Islamabad, Pakistan; 6 Institute of Translational Medicine, Zhejiang Shuren University, Hangzhou, 310022 China

**Keywords:** Stem cell-like macrophages, Trained immunity, Bcl6, Memory-like macrophages

## Abstract

Cancer development is a long-lasting process during which macrophages play a pivotal role. However, how macrophages maintain their cellular identity, persistence, expanding and pro-tumor property during malignant progression remains elusive. Inspired by the recent report of the activation of stem cell-like self-renewal mechanism in mature macrophages, we postulate that intra-tumoral macrophages might be trained to assume stem-like properties and memory-like activity favoring cancer development. Herein we demonstrated that tumor infiltrating macrophages rapidly converted into the CD11b^+^F4/80^+^Ly6C^−^Bcl6^+^ phenotype, and adopted stem cell-like properties involving expression of stemness-related genes, long-term persistence and self-renewing. Importantly, Bcl6^+^ macrophages stably maintained cell identity, gene signature, metabolic profile, and pro-tumor property even after long-term culture in tumor-free medium, which were hence termed stem cell-like memory macrophages (SMMs). Mechanistically, we showed that transcriptional factor Bcl6 co-opted the demethylase Tet2 and the deacetylase SIRT1 to confer the epigenetic imprinting and mitochondrial metabolic traits to SMMs, bolstering the stability and longevity of trained immunity in tumor-associated macrophages (TAMs). Furthermore, tumor-derived redHMGB1 was identified as the priming signal, which, through TLR4 and mTOR/AKT pathway, induced Bcl6-driven program underpinning SMMs generation. Collectively, our study uncovers a distinct macrophage population with a hybrid of stem cell and memory cell properties, and unveils a regulatory mechanism that integrates transcriptional, epigenetic and metabolic pathways to promote long-lasting pro-tumor immunity.

**Supplementary Information:**

The online version contains supplementary material available at 10.1007/s00018-022-04660-0.

## Introduction

The organism evolves to remember the initial encounters and mount a rapid and enhanced response upon re-stimulation, which is termed immunological memory. It has long been held that immunological memory only occurs in adaptive immune cells such as T and B lymphocytes, but emerging evidences demonstrate that innate immune cells, especially macrophages, are also capable of inducing recall responses, which is referred as trained immunity [1, 2]. Microbial components such as β-glycan, lipopolysaccharide (LPS), vesicular stomatitis virus (VSV) and Staphylococcus aureus (*S. aureus*) have been reported to induce trained immunity in macrophages through rewiring metabolic and epigenetic pathways [3–5]. Under these circumstances, the priming signals driving memory macrophages generation are mostly from pathogen-associated molecules and sensed by pattern-recognition receptors (PRRs) on cellular surfaces. Of interest, PRRs on macrophages recognize not only invading agents but also endogenous signals involving damage-associated molecules, metabolic byproducts and cytokines [6–8]. Compared with current knowledge about infection-induced innate immune memory, our understanding of the trained immunity that is caused by non-infectious cues like tumor-derived signals, is relatively scarce.

To establish durable immunological memory, immune cells require to live long, stably retain “primed” phenotype, and readily induce recall responses upon re-encountering of the relevant stimuli. In adaptive immune systems, T or B lymphocytes exploit the TCR/BCR-driven clonal selecting machinery to generate antigen-specific population, and a small subpopulation are committed to reconstitute memory cell compartment. By contrast, mature macrophages, except for some tissue-resident macrophages, generally have short half-life and are unable to induce memory-like responses [9, 10]. The concept however is challenged as emerging evidences show that differentiated macrophages can also maintain endurable immunological memory and induce the so called trained immunity or innate immune memory [5, 6, 11–16]. Of interest, recent studies demonstrate that trained immune cells adopt a hybrid of stem and memory cell status, which may set a basis for stability and longevity of immunological memory. Stemness-related properties such as self-renewing and reconstituting capability have been implicated in the development of immune memory cells. T stem cell memory cells (T_SCM_), for instance, are defined as stem-like cell lineage with self-expanding, long-living, and apoptosis-resistant properties [15]. CD8 T memory cells can also develop into stem-like cells through re-activation of naïve-associated genes for de-differentiation [16]. Similarly, tissue resident macrophages are reported to share with their progenitors a network of stem-related genes, though controlled by lineage-specific factors [17]. Under this circumstance, the transcriptional factors MafB and c-MAF prove to restrain the expression of stem-related genes in alveolar macrophages (AMs) and peritoneal macrophages respectively, and down-regulation of them would lead to de-repression of multipotent and self-renewing genes in mature macrophages [18]. Likewise, the transcription factors Bhlhe40 and Bhlhe41 were recently reported to control the activation of self-renewing genes and govern long-living and homeostasis of pulmonary macrophages [19]. B cell lymphoma 6 (Bcl6) is a master transcriptional regulator with the ability to regulate a wide range of genes essential for cellular differentiation, stemness, survival and proliferation. It plays a key role in regulating differentiation of Tfh cells, germinal center B cells and other immune cells such as dendritic cells and neutrophils [20–22]. More recent data indicate that Bcl6 is required for differentiation of memory T and B cells, stem cell-like memory cells particularly [23–25], but its role in memory macrophages or trained immunity generation has never been explored.

Trained immunity is orchestrated by the regulatory program integrating transcriptional epigenetic and metabolic pathways. Metabolic pathways, as an essential source for bioenergetics, brick components and epigenetic co-factors, promote functional differentiation, long-term persistence and expanding of trained immune cells, especially in generally nutrients-stressed tumor microenvironment. Although evidences have demonstrated that glycolytic and mevalonate metabolism played a critical role in promoting trained immunity in human monocytes [26], other studies revealed that mitochondrial function and metabolism was indispensable for trained program in myeloid cells [27, 28]. It is currently recognized that, beyond as energy suppliers, mitochondria also produce metabolic intermediates involving α-ketoglutarate (αKG), NAD^+^ and acetyl-coA, which would serve as the substrates and co-factors of chromatin modifying enzymes Tet2, SIRT1 and P300/CBP to mediate metabolic-epigenetic regulation of memory cells [29, 30]. However, till now we know little about the metabolic-epigenetic mechanism that drives the formation of trained immunity during tumorigenesis.

In the present study, we unravel a distinct population of tumor-associated macrophages (TAMs), which were featured with long-tern persistence, self-renewing potential, stem-related gene expression, and more importantly, retaining tumor-promoting activity even after long-term culture in the absence of tumor stimulation. The findings therefore lead to the terminology of stem-like memory macrophages (SMMs). We further demonstrated that Bcl6 played a central role in coordinating the transcriptional, epigenetic and metabolic pathways to control SMMs commitment underpinning cancer progression. Moreover, reduced but not oxidative HMGB1 was identified as the priming signal to be sensed by TLR4 and mTOR pathway to induce Bcl6-driven program. The findings provide a novel insight into tumor-elicited innate immune memory, opening a new avenue for cancer immunotherapy targeting stem-like memory macrophages.

## Results

### TLR4-expressing macrophages promote cancer progression.

**Fig. 1:**
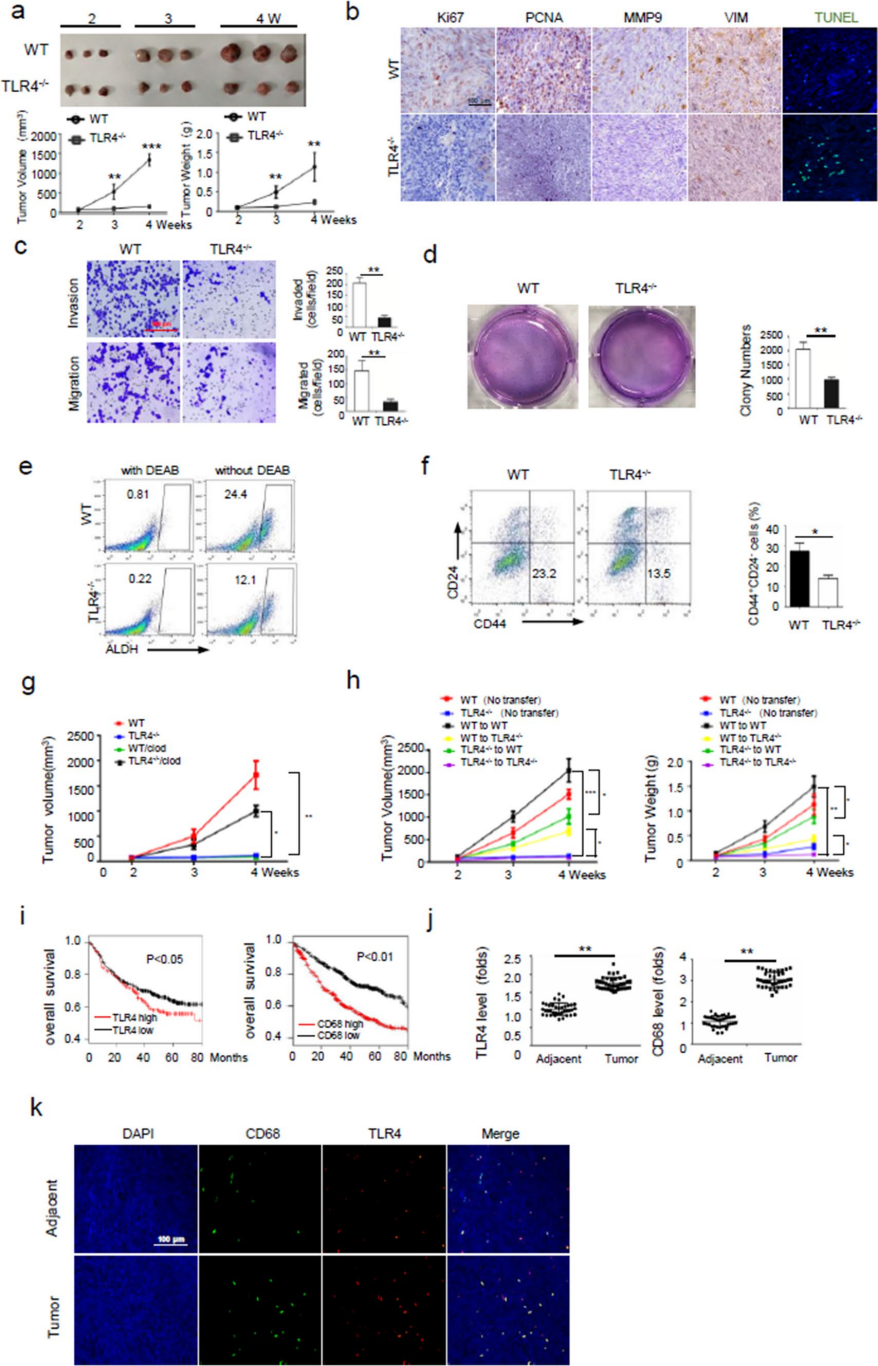
TLR4-expressing macrophages promote cancer progression.** a** The volumes and weights of tumor developed in WT and TLR4^−/−^ mice transplanted with LLCs (*n* = 6, s.c.). Shown are representative results from 3 independent experiments. **b** Representative images showing immunohistochemical staining of malignancy-associated molecules as indicated in murine tumor sections. DAPI, nucleus. **c**, **d** The invasive and migratory capability (c), and cellular colonigenic ability (d) of tumor cells. **e**, **f** Flow cytometry of the frequencies of ALDH^+^ or CD44^+^CD24^–^ subpopulation in tumors. DEAB was used as a negative control to set the gates. **g** Tumors volumes were assessed in mice with macrophages depletion by clodronate (clod) treatment; control liposome was also applied. **h** Tumor volumes were measured in LLC-implanted mice that adoptively received macrophages from donator mice as indicated (left); The histogram showing average tumor weights at the end of study (right). **i** Kaplan–Meier survival analysis of the association of TLR4 and CD68 levels with the overall survival of patients with lung adenocarcinoma based on the TCGA database. *p* < 0.05 or 0.01 by log-rank test. **j** qPCR assay of TLR4 and CD68 levels in cancerous or adjacent tissues from NSCLC patients (*n* = 37). **k** Immunofluorescent staining of TLR4 and CD68 in cancerous or adjacent tissues from NSCLC patients. Representative images from similar results are shown. The data from 2–3 independent experiments are expressed as means ± SEM. *p < 0.05, **p < 0.01, ***p < 0.001 by student’s* t* test

To investigate whether and how trained immunity was formed during cancer development, we established a subcutaneous Lewis lung carcinoma (LLC) model in mice that were devoid of TLR4, the prototypic pattern-recognition receptor for both pathogen and damage-associated signals. Strikingly, our data demonstrated that compared with TLR4^−/−^ mice, wild type (WT) mice developed remarkably greater tumors (Fig. 1a). Moreover, tumors developed in WT mice displayed more aggressive properties, as manifested by increased expression of molecules representing cell proliferation (Ki67, PCNA), invasion (MMP9) and mesenchyme origin (vimentin), and decreased apoptotic signal (TUNEL) (Fig. 1b, Fig. S1a). Consistently, cancer cells from WT mice exhibited enhanced migratory, invasive, proliferative ability, and increased resistance to chemotherapeutic agent (Fig. 1c, d; Fig. S1b). The percentages of aldehyde dehydrogenase (ALDH)^+^ or CD44^+^CD24^−^, indicative of stem-related property, were increased in tumors formed in WT mice compared with that in TLR4^−/−^ animals (Fig. 1e, f). Along with this, the abundance of intratumoral cytotoxic T cells (CTLs), particularly interferon-γ-producing CTLs, was reduced, while the proportion of regulatory T cells was elevated in WT relative to TLR4^−/−^ mice (Fig. S1c-e). Collectively, our data demonstrated that TLR4 played a non-intrinsic role in promoting cancer progression, which was likely associated with compromised anti-tumor immunity.

TLR4 is one of the best-characterized immune receptors preferentially expressed in macrophages and other myeloid cells. Considering that tumor niches were frequently populated with macrophages, a key player in tumor immunity, we postulated that intratumoral macrophages were related with differential tumor growth in TLR4 sufficient or deficient mice. To test this, we applied clodronate-encapsulated liposomes to deplete tumor-associated macrophages (TAMs) (Fig. S1f). The result showed that removal of TLR4^+/+^ macrophages significantly reduced tumor sizes, whereas depletion of TLR4^−/−^ macrophages increased tumor mass (Fig. 1g). To further confirm this, we then adoptively transferred the in vitro differentiated bone marrow-derived macrophages (BMDMs) to LLC-implanted mice. Similarly, delivery of TLR4^+/+^ macrophages increased tumor burdens in both WT and TLR4^−/−^ mice, while transfer of TLR4^−/−^ macrophages yielded the opposite effect (Fig. 1h). Thus, our data consistently demonstrated that TLR4 proficient macrophages promoted while TLR4 deficient macrophages impeded tumor progression. In support of this, the TCGA-based analysis revealed that the levels of human macrophage marker CD68 and TLR4 were negatively correlated with the overall survival of patients with lung cancer (Fig. 1i). In parallel, TLR4 and CD68 were abundantly expressed and co-localized in cancerous areas but not adjacent tissues in samples from lung cancer patients (Fig. 1j, k; Fig. S1g). Taken together, our data demonstrated that TLR4-expressing macrophages played a key role in promoting cancer progression.

### Tumors foster the generation of stem-like memory macrophages (SMMs) through TLR4 pathway

**Fig. 2:**
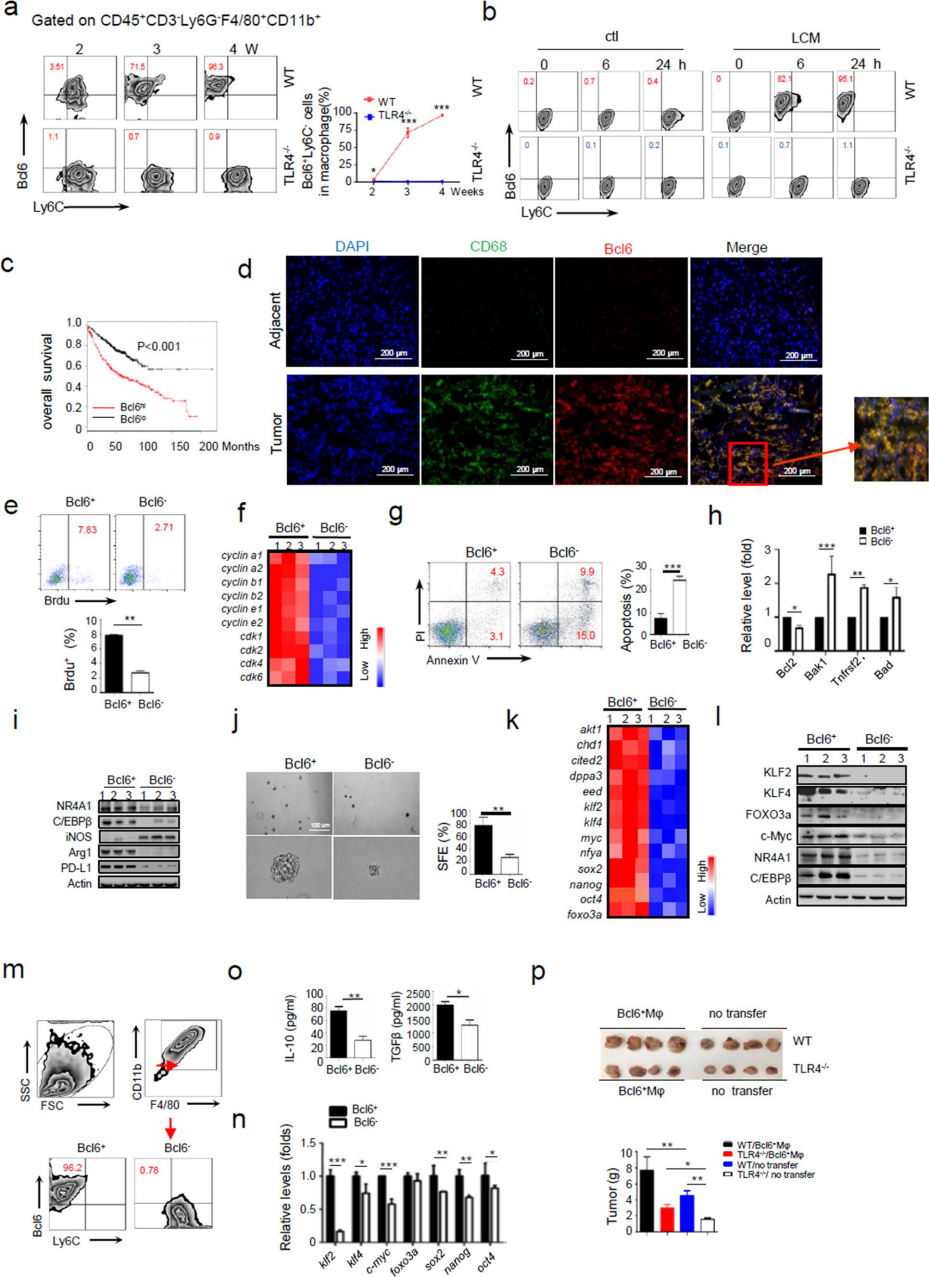
Tumors foster the generation of stem-like memory macrophages through TLR4 pathway. **a** Flow cytometry of macrophages in tumors developed in WT and TLR4^−/−^ mice at the indicated time periods post LLC inoculation. CD45^+^ leukocytes were identified, doublets excluded by FSC-W vs. FSC-A and dead cells excluded by DAPI. Live cells were then gated on CD3^−^Ly6G^−^F4/80^+^CD11b^+^ for macrophages and further stratified by Ly6C and Bcl6 level. **b** Bone marrow-derived macrophages (BMDMs) were incubated with fresh medium alone (Ctl) or LLC-conditioned medium (LCM) for the indicated time periods. Flow cytometry of Bcl6^+^CD11b^+^F4/80^+^ macrophages. **c** Kaplan–Meier survival analysis of the association of Bcl6 levels with the overall survival of patients with lung adenocarcinoma based on the TCGA database. *p* < 0.001 by log-rank test. **d** Co-localization of CD68 and Bcl6 on cancerous or adjacent tissues from cancer patients. Shown are representative images. **e**-**l** Bcl6^+^ and Bcl6^−^ macrophages were sorted from TLR4^+/+^ and TLR4^−/−^ mice respectively at 24 days post LLC inoculation as described in Methods, and subjected to functional analysis. BrdU-incorporating test of cellular proliferative capability (**e**); heatmapping of cell cycle-related genes (**f**); Flow cytometry of apoptotic events (**g**) and qPCR analysis of apoptosis-related genes (**h**); Immunoblotting of the lineage-associated molecules (**i**); Spheroid-forming assay of cellular self-renewing capability (**j**); qPCR analysis (**k**) and immunoblotting (l) of stem-related genes. Bcl6^+^ and Bcl6^−^ TAMs (**i**, **l**) or TLR4^+^ and TLR4^−^ TAMs (**e**–**h**, **j**, **k** herein representing Bcl6^+^ and Bcl6^−^ TAMs) were applied. **m**–**o** Sibling cells were recollected from Bcl6^+^ spheroids cultured in tumor-free medium for 5 weeks and subjected for functional analysis. Flow cytometry of the frequency of Bcl6^+^ SMMs (**m**); qPCR analysis of stem-related genes (**n**); ELISA assay of IL-10 and TGF-β (**o**). **p** Size and weights of tumors developed in WT or TLR4^−/−^ mice receiving Bcl6^+^ spheroid-generating cells or not. Shown are representative images and the data from three independent experiments are expressed as means ± SEM. *p < 0.05, **p < 0.01, ***p < 0.001 by student’s *t* test

To further define the role of TLR4^+/+^ macrophages in cancer development, we then isolated and characterized the intratumoral macrophages, typically defined as CD45^+^Ly6G^−^CD11b^+^F4/80^+^CD3^−^ (Fig. S2a, b). The phenotypic analysis revealed that WT but not TLR4^−/−^ macrophages exhibited a marked decline in expression of Ly6C, a proinflammatory monocyte marker [31]. Of note, the level of Bcl6, a transcriptional regulator key for immune cell differentiation, was significantly elevated in TLR4^+/+^ macrophages. By contrast, TLR4^−/−^ macrophages remained Bcl6^low^Ly6C^high^ phenotype during the whole period of tumorigenesis, implying that induction of TLR4 and subsequent Bcl6 were associated with macrophage tumor-promoting activity (Fig. 2a). Supportively, the in vitro culture showed that treatment of LLC-conditioned medium (LCM) caused the transformation of TLR4^+/+^ but not TLR4^−/−^ macrophages to Bcl6 ^high^Ly6C^low^phenotype (Fig. 2b, Fig S2c-e).

To further confirm these findings, we also established a murine orthotropic model by intrapulmonary implantation of LLC cells. Similarly, more progressive tumors, along with augmented percentage of Bcl6^high^ macrophages, were observed in WT mice relative to TLR4^−/−^ littermates (Fig. S3a-c). The data thus collectively indicated that TLR4-mediated Bcl6 induction was essential for macrophages differentiation underpinning tumor progression. To be clinically relevant, co-localization of Bcl6 and CD68^+^ was detected in lung cancer samples, which was correlated with poor outcome of cancer patients (Fig. 2c, d).

Inspired by the above findings, we conducted a more detailed analysis of Bcl6^+^ macrophages fostered by tumors. By bromodeoxyuridine (BrdU)-labeling method, we revealed that Bcl6^+^ macrophages were more proliferative than Bcl6^−^ subset, with concomitantly increased expression of cycle-related molecules (Fig. 2e, f). Also, Bcl6^+^ subset exhibited apoptosis-resistant property with up-regulation of anti-apoptotic molecules expression but decreased levels of pro-apoptotic factors (Fig. 2g, h). Moreover, the gene profiling revealed that, compared with their counterparts, Bcl6^+^ macrophages expressed higher levels of NR4A1 and C/EBPβ (Fig. 2i), the transcriptional factors involved in myeloid differentiation and monocyte/macrophage conversion [31, 32]. Additionally, Bcl6^+^ cells demonstrated to preferentially express the immune suppressive molecules such as PD-L1, Arg1, IL-10 and TGFβ while restraining the expression of pro-inflammatory cytokines including iNOS, IL-6 and TNFα (Fig. 2i; Fig. S4a, b). Together, our data indicated that tumor-elicited Bcl6^high^ macrophages were proliferative, surviving and immune suppressive.

Trained immunity is a process requiring innate immune cells to remember and maintain the imprinters given by initial stimuli over a long period of time. Given the rapid induction and persistence of Bcl6^+^ macrophages during tumor development, we next explored whether this subpopulation would stably retain the phenotype and functionality initially formed. For this, Bcl6^+^ macrophages were isolated from tumor-bearing mice and cultured in vitro in tumor-free medium. Interestingly, we observed that Bcl6^+^ macrophages persisted for at least 6 weeks in the in vitro culture with no tumor signaling input. Moreover, the cells manifested robust spheroid-forming capability, indicative of stem cell-like self-renewing potential (Fig. 2j), and consistently expressed stem-related genes such as *klf2, klf4, myc, Sox2, nanog* and *oct4, *etc*.* (Fig. 2k,l). The data thus indicated that Bcl6^+^ macrophages, once impinged by tumors, were able to persist and proliferate by self-renewing, similar to stem cells.

Since self-renewing is generally thought as a prerequisite for maintaining stability and expanding of memory cells, we therefore processed to examine whether Bcl6^+^ macrophages possessed the memory-forming potential. To this end, functional analysis was conducted on Bcl6^+^ sibling cells from TAMs spheroids. Impressively, the results showed that, even after long-term culture in tumor-free medium, the spheroid cells still retained Bcl6^high^ phenotype, stem-related gene signature and immune-suppressive cytokine profile, as their progenitors (Fig. 2m-o). More strikingly, the sibling cells, when adoptively transferred to LLC-implanted mice, yielded remarkably greater tumor (Fig. 2p). The findings indicated that descendants of Bcl6^+^ macrophages stably retained the phenotypic and functional properties formed in tumor niches. Interestingly, we additionally observed that delivery of Bcl6^+^ spheroid cells to endotoxic mice induced by lipopolysaccharide (LPS), a prototypic ligand of TLR4, led to alleviated inflammatory pathology and improved survival (Fig. S4c, d). The results supported the stability and relative specificity of trained immunity in macrophages. We also incubated Bcl6^+^TAMs with LPS /IFNɣ, and found that Bcl6^+^ macrophages still expressed the immune-tolerant markers such as Arg1, Mrg1, Mgl2, IL-10 and TGF-β, as well as stem-related genes including *klf2, klf4, myc and foxo3a* (Fig. S4e, f). In line, the self-renewing ability, as demonstrated by spheroid-forming property, was retained in these cells (Fig. S4g). However, we noted that the expression of pro-inflammatory cytokines such as iNOS, IL-12, IL-6, IL-1β was increased upon LPS/IFNɣ stimulation (Fig. S4h). Thus, it appears that Bcl6^+^ TAMs, to an extent, retain the plasticity and can be manipulated for immunotherapy, but further studies are needed to clarify this issue. Taken together, our data indicated that tumor-elicited Bcl6^+^ macrophages adopted a hybrid status of stem and memory cells to maintain phenotypic and functional features initially impinged by tumors. We therefore tentatively named Bcl6^+^ TAMs as stem-like memory macrophages (SMMs).

### Mitochondrial metabolism supports metabolic fitness of SMMs during tumor development

**Fig. 3:**
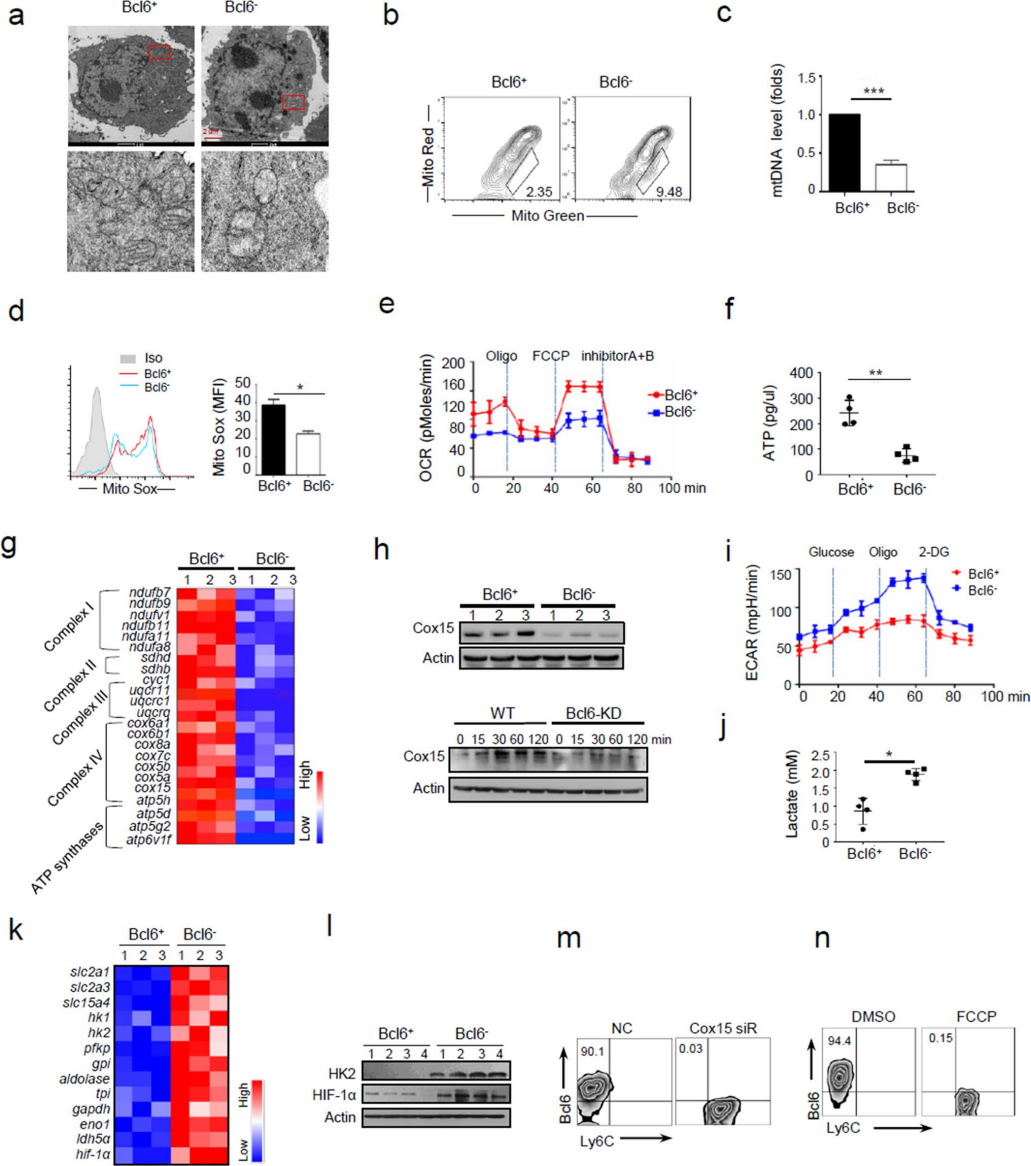
Mitochondrial metabolism supports SMMs metabolic fitness during tumor development. **a**-**g** Bcl6^+^and Bcl6^−^ TAMs were sorted from TLR4^+/+^ and TLR4^−/−^ mice respectively at 24 days post LLC inoculation as described in Methods, and subjected to metabolic characterization. Representative transmission electron micrographs (TEM) showing mitochondrial amount, morphology and cristae (**a**); Flow cytometry of dysfunctional mitochondria by staining with MitoTracker Red and MitoTracker green (**b**); qPCR analysis of mitochondrial DNA (**c**); Flow cytometry and quantification of mitochondrial ROS levels by staining with MitoSox (**d**); Oxygen consumption rate (OCR) levels (**e**); ATP generation (f); Heatmapping of mitochondrial respiratory-associated genes (**g**); **h** Immunoblotting of Cox15 in Bcl6^+^ and Bcl6^−^ macrophages (upper), or in WT or Bcl6-knockdown (KD) macrophages treated with LCM for the indicated time periods (lower); **i**-**l** Extracellular acidification rate (ECAR) (**i**), lactate acid level (**j**), heatmapping of glycolytic genes (**k**), and immunoblotting of HK2 and HIF-1α (**l**) in Bcl6^+^ and Bcl6^−^ macrophages. The sorted Bcl6^+^ and Bcl6^−^ TAMs (**a**, **h**, **l**) or TLR4^+^ and TLR4^−^ TAMs (**b**-**g**, **i**-**k** herein representing Bcl6^+^ and Bcl6^−^ TAMs) were applied. **m**, **n** The percentages of Bcl6^+^ SMMs (gated on CD45^+^Ly6C^−^CD11b^+^F4/80^+^) generated from LCM-conditioned macrophages that were either transfected with Cox15-specific or non-specific (NC) siRNA, or pre-treated with FCCP or DMSO. Shown are representative images. The data from three independent experiments are expressed as means ± SEM. *p < 0.05, **p < 0.01, ***p < 0.001 by student’s *t* test

As long-living memory macrophages is heavily dependent on metabolic fitness particularly in nutrient-stressed tumor milieu [33], we then analyzed the metabolic characteristics of Bcl6^+^ SMMs. The two major energy-supplying pathways, namely, mitochondrial metabolism and aerobic glycolysis, were therefore profiled in isolated TAMs. Indeed, Bcl6^+^ macrophages displayed more quantities of mitochondria, which were featured with healthy rod-shape and organized cristae. Bcl6^−^ cells nevertheless harbored dysfunctional mitochondria with swollen shape and irregular rarefied cristae (Fig. 3a). Congruently, Bcl6^+^ macrophages exhibited more functional mitochondria (as stained by Mito-Green/Red), higher mtDNA level and increased mROS release (Fig. 3b-d). Associated with this, oxygen consumption rate (OCR), ATP generation, as well as expression of mitochondrial respiratory molecules were elevated in Bcl6^+^ macrophages (Fig. 3e-h). By contrary, Bcl6^−^ macrophages were more dependent on anaerobic glycolysis, as depicted by their elevation in extracellular acid rate (ECAR), lactate production and expression of glycolysis-related genes (Fig. 3i-l). The data thus indicated that Bcl6^+^ macrophages preferentially assumed mitochondria- metabolism and restrained glycolytic pathway, ensuring their metabolic fitness during competing with ever-growing tumor cells, a glycolysis-favoring population. As an additional support, we showed that disruption of mitochondrial respiratory chain by deletion of Cox15, an assembly factor of mitochondrial respiratory complex IV, or treatment of trifluoromethoxy carbonylcyanide phenylhydrazone (FCCP), the uncoupler of mitochondrial oxidative phosphorylation, led to an almost abrogation of Bcl6^+^ macrophages (Fig. 3m, n). The data underscored the importance of mitochondrial integrity and metabolism in promoting SMM generation and maintenance. Together, our data indicated that Bcl6^+^ TAMs adopted mitochondria-dominant metabolism to support their metabolic adaptiveness in nutrient-stressed tumor niches.

### Bcl6 is necessary and sufficient for the development of SMMs instructed by tumor

**Fig. 4:**
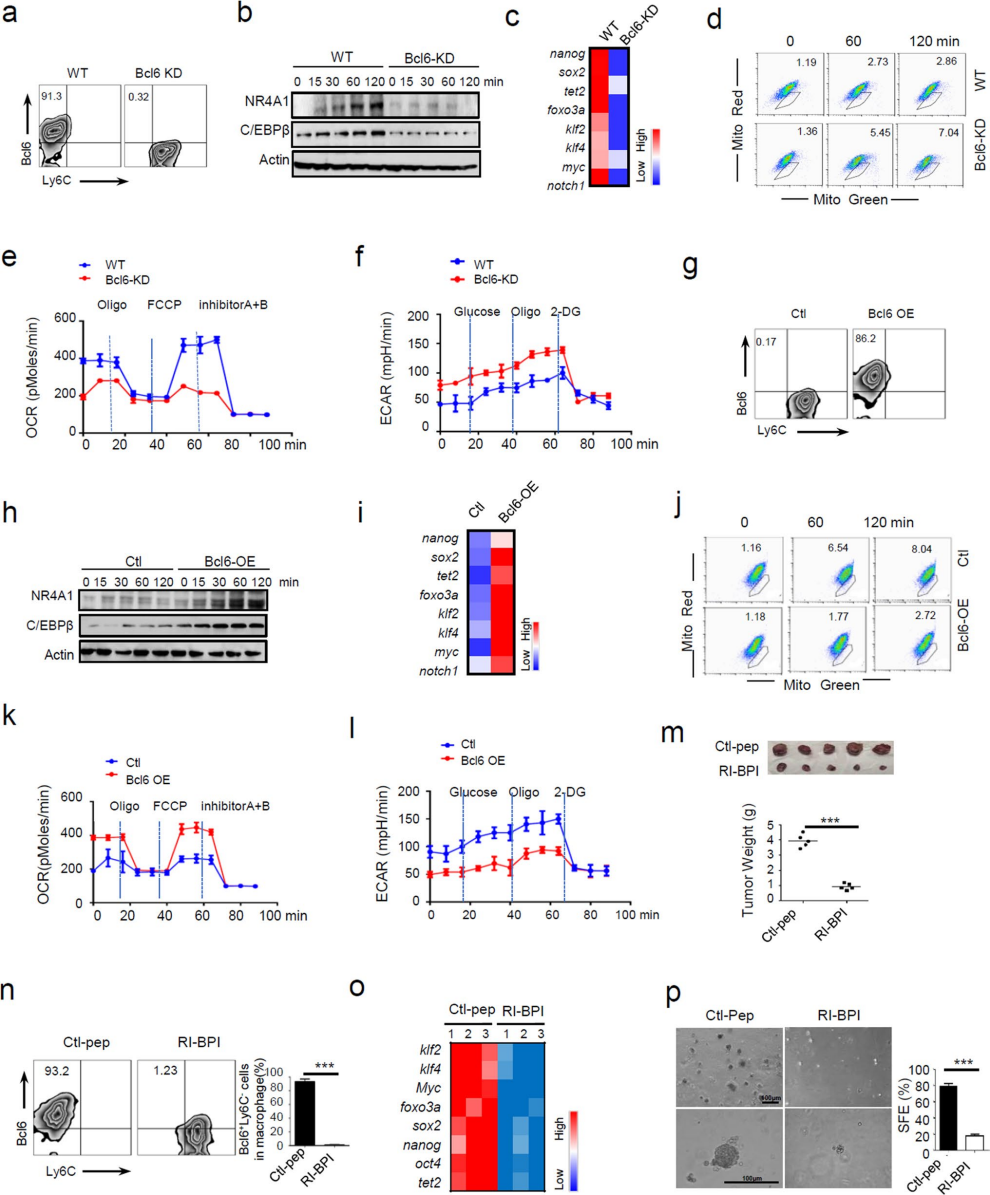
Bcl6 is necessary and sufficient for the development of SMMs instructed by tumor. **a**-**f** Macrophages were generated from BMDMs with or without Bcl6 knockdown (KD), followed by LCM stimulation for 24 h or the indicated time periods. Flow cytometry of the frequency of Bcl6^+^ cells (**a**); Immunoblotting of NR4A1 and C/EBPβ (**b**); Heatmapping of stem-related genes (**c**); Flow cytometry of mitochondria by staining with MitoTracker Red and MitoTracker green (**d**); Measurement of OCR and ECAR levels by Seahorse (**e**, **f**). **g**-**l** Macrophages were generated from TLR4^−/−^ BMDMs that were transfected with Bcl6-rexpressing (Bcl6-OE) or control ones, followed by LCM stimulation for 24 h or the indicated time periods. Flow cytometry of the frequency of Bcl6^+^ SMMs (**g**); Immunoblotting of NR4A1 and C/EBPβ (**h**); Heatmapping of stem-related genes (**i**); Flow cytometry of mitochondria upon staining with MitoTracker Red and MitoTracker green (**j**); Measurement of OCR and ECAR levels (**k**, **l**). **m**-**p** Tumors developed in LLC-inoculated mice (*n* = 5) that were co-administrated with RI-BPI or control peptide (500ug/mice, i.p.). Size and weight of tumors (**m**); Flow cytometry of Bcl6^+^ SMMs (**n**). Heatmapping of stem-related genes (**o**), and the spheroid-forming ability of TAMs (**p**). Shown are representative images, and SFE was evaluated as the percentage of seeded cells generating a spheroid. The data from three independent experiments are expressed as means ± SEM. *p < 0.05, **p < 0.01, ***p < 0.001 by student’s *t* test

Next, to further dissect the role of Bcl6 in SMMs differentiation, we specifically knocked down Bcl6 in murine macrophages, which were then culture in LLC-conditioned medium (LCM). Strikingly, Bcl6 deletion inhibited the generation of SMMs upon LCM stimulation, as demonstrated by blunted expression of lineage-defining factors (NR4A1, C/EBPβ) and stem-related genes, as well as increased amounts of dysfunctional mitochondria (Fig. 4a-d). In parallel, Bcl6 ablating cells exhibited decreased OCR level but heightened ECAR rate, with insufficient ATP generation and augmented lactate production (Fig. 4e, f; Fig. S5a, b). In addition, we obtained myeloid Bcl6 knockout (Bcl6^∆LysM^) mice from other investigator’s lab and validated major of our findings. The results consistently demonstrated that myeloid ablation of Bcl6 remarkably precluded tumor growth in a murine subcutaneous LLC model, accompanied by the loss of Bcl6^+^ TAMs, a subpopulation featured with self-renewing potential, stem-related gene expression, mitochondria-dominant metabolism and pro-tumor property (Fig. S5c-h). The results thus indicated that Bcl6 was required for stem-like macrophages generation instructed by tumors, deletion of which diverted macrophage differentiation into the opposite subset.

We next sough to address whether resumption of Bcl6 would restore SMM generation in TLR4^−/−^ macrophages, the population proved to be unable to induce Bcl6 upon tumor stimulation (Fig. 2a). Indeed, the data showed that enforced expression of Bcl6 enabled TLR4^−/−^ macrophages to take on SMM-like features, as demonstrated by increased expression of lineage- and stemness-associated genes, improved mitochondrial potential, elevated mitochondrial respiration and ATP generation, as well as reduced levels of ECAR and lactate release (Fig. 4g-l; Fig. S5i, j). We thus concluded that Bcl6 was required and sufficient for the development of SMMs instructed by tumor-derived signals. In addition, we exploited Bcl6 inhibitory peptide (RI-BPI) [34]-harboring nanoparticles to assess the in vivo impact of Bcl6 inhibition in LLC-implanted mice (Fig. S5k). The results showed that, compared with control nanoparticles, administration of RI-BPI-nanoparticles profoundly reduced tumor burdens (Fig. 4m). Along with this, the differentiation of Bcl6^+^ SMMs was hindered, as evidenced by reduced expression of stem-related genes and impaired self-renewing potential (Fig. 4n-p).

Collectively, our data indicated that Bcl6 played a central role in governing SMMs commitment and hence tumor progression, making it a potential target for cancer immunotherapy by interfering trained immunity.

### Bcl6 coordinates transcriptional and metabolic programs underpinning SMMs generation

**Fig. 5:**
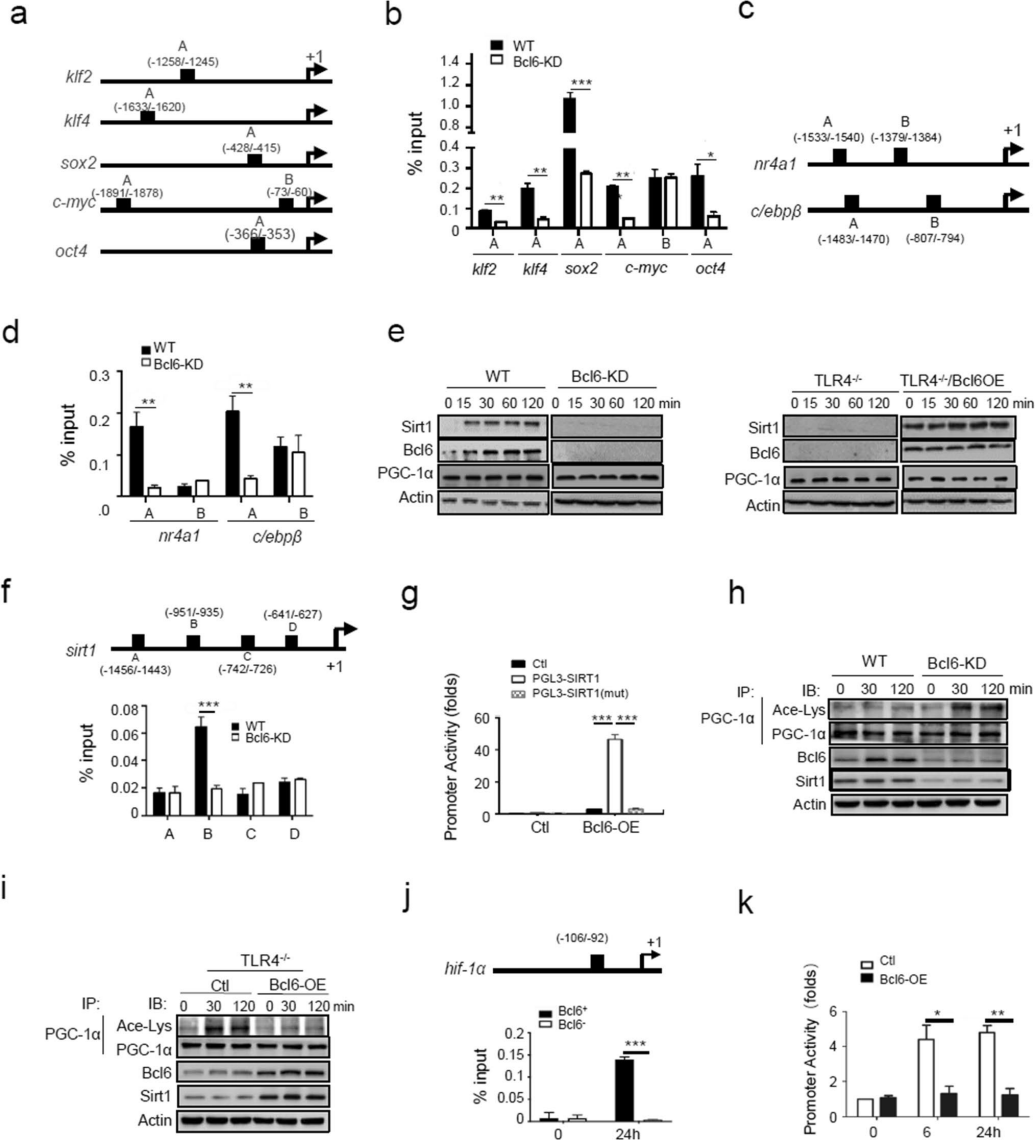
Bcl6 coordinates transcriptional and metabolic programs underpinning SMMs generation. **a** The putative binding sites of Bcl6 at the loci of stem-related genes. **b** Chromatin immunoprecipitation (ChIP) assay of Bcl6 enrichment at the loci of stem-related genes. **c** The putative binding sites of Bcl6 at the loci of lineage-associated genes (*nr4a1* and *c/ebpβ*). **d** ChIP assay of Bcl6 enrichment at the loci of lineage-related genes in Bcl6-knockdown (Bcl6-KD) and control macrophages. **e** Immunoblotting of SIRT1 and PGC-1α in Bcl6-KD and control macrophages, or in TLR4^−/−^ macrophages that were transfected with Bcl6-expressing (Bcl6-OE) or control plasmids. **f** The putative binding sites of Bcl6 at the *sirt1* promoter region (upper), and ChIP analysis of Bcl6 enrichment at the *sirt1* locus (lower). **g** The SIRT1-driven promoter activity detected in RAW 264.7 cells transfected with pGL3-SIRT1, mutant pGL3-SIRT1 (mut) or control plasmids respectively, along with or without Bcl6-expressing plasmids. **h** Co-immunoprecipitation (Co-IP) of acetylated and total PCG1-α in Bcl6-KD or control macrophages upon LCM stimulation for the indicated time periods. **i** Co-IP of acetylated and total PCG1-α in TLR4^−/−^ macrophages that were transfected with Bcl6-OE or control plasmids, followed by LCM stimulation for the indicated time periods. **j** The putative binding sites of Bcl6 at the promoter region of *hif-1α* (upper), and ChIP analysis of Bcl6 enrichment at the *hif-1α* locus in Bcl6^+^ or Bcl6^−^ macrophages (lower). **k** The HIF-1α-dependent promoter activity detected in RAW 264.7 cells that were co-transfected with pGL3-HIF-1*α* plasmids and Bcl6-expressing plasmids, followed by LCM stimulation for the indicated time periods. Shown are representative images and the data from three independent experiments are expressed as means ± SEM. *p < 0.05, **p < 0.01, ***p < 0.001 by student’s *t* test

Bcl6 is known as a multifunctional factor with the ability to enhance or repress gene expression depending on its association with distinct cofactors in a given context. We next explored how Bcl6 governed SMMs differentiation by firstly assessing its regulation of stem- and lineage-related genes. Indeed, the bioinformatics analysis revealed an enrichment of Bcl6-binding motifs in 5’UTR of stem-associated genes such as *Klf2, Klf4, Sox2, c-myc* and *oct4*, as well as that of lineage-related factors *Nr4a1 and C/ebpβ* (Fig. 5a, c). Chromatin immunoprecipitation (CHIP) assays confirmed that Bcl6 specifically bond to the loci of these genes, and deletion of Bcl6 abolished the associations (Fig. 5b, d). The results, combined with the observed transcriptional regulation of the relevant genes (Fig. 4b, c, h, i), pointed to Bcl6 as a genuine transcriptional factor for SMM-related genes.

We next investigated how Bcl6 orchestrated the metabolic pathway that determined SMMs fitness, specifically, how it reinforced mitochondrial metabolism while restraining glycolytic pathway in macrophages. We initially examined the effect of Bcl6 on the expression of peroxisome proliferator-activated receptor-gamma coactivator-1α (PGC-1α), the factor key for mitochondrial biogenesis and metabolism. Intriguingly, either knockdown or overexpression of Bcl6 exerted marginal effect on PGC-1α level, implying that Bcl6 did not directly regulate gene expression. Nevertheless, we noted that the level of sirtuin1 (SIRT1), an NAD^+^-dependent deacetylase, was repressed by Bcl6 deletion and increased upon Bcl6 overexpression, suggesting the modulation of SIRT1 by Bcl6 (Fig. 5e). Supportively, the bioinformatics analysis revealed 4 putative Bcl6 binding sites at 5’UTR of *Sirt1* gene, among which B site (-951 ~ -935) was confirmed by Co-IP (Fig. 5f). The luciferase assay consistently demonstrated that SIRT1-driven promoter activity was induced by Bcl6 in a site-specific manner (Fig. 5g). The results thus substantiated the transcriptional regulation of the deacetylase SIRT1 by Bcl6.

It has been reported that the optimized activity of PGC-1α required the SIRT1 to remover acetyl groups from it [35], which promoted us to further investigate whether Bcl6 modulated PGC-1α activity through regulating the level of SIRT1. Indeed, correlating with reduced SIRT1 level, elevated acetylation and hence compromised PGC-1α activity were induced upon Bcl6 knockdown (Fig. 5h). Conversely, restoration of Bcl6 expression elevated the expression of SIRT1 in TLR4^−/−^ macrophages, leading to de-acetylation and hence activation of PGC-1α (Fig. 5i). To be functionally relevant, SIRT1 overexpression remarkably improved mitochondrial function and ATP production, whereas SIRT1 deficiency impaired mitochondrial metabolism (Fig. S6a-d). The data thus supported that Bcl6 acted through the SIRT1/PGC-1α axis to boost mitochondrial metabolism in SMMs.

As depicted above, differentiation of SMMs was a coherent process coupling enhanced mitochondrial metabolism with restrained glycolytic catabolism, we thus also explored the mechanism underlying Bcl6-mediated regulation of glycolytic pathway in tumor-conditioned macrophages. Of interest, a putative binding site of Bcl6 was identified within 5’ UTR of *Hif-1α*, a pace-limiting factor of glycolytic pathway (Fig. 5j). The dual-fluorescence reporter assay confirmed that Bcl6 specifically repressed HIF-1α-driven promoter activity (Fig. 5k). These results, along with the repression of glycolytic genes by Bcl6 observed above (Fig. 3k, l), led to the conclusion that Bcl6 negatively regulated the transcription of glycolytic genes. Taken together, our study indicated that Bcl6 coupled transcriptional and post-translational mechanisms to regulate the expression of stem-, lineage- and metabolism-related genes, facilitating cellular differentiation and metabolic adaptiveness of SMMs.

### Bcl6 co-opts the demethylase Tet2 to shape SMMs epigenetic signature

**Fig. 6:**
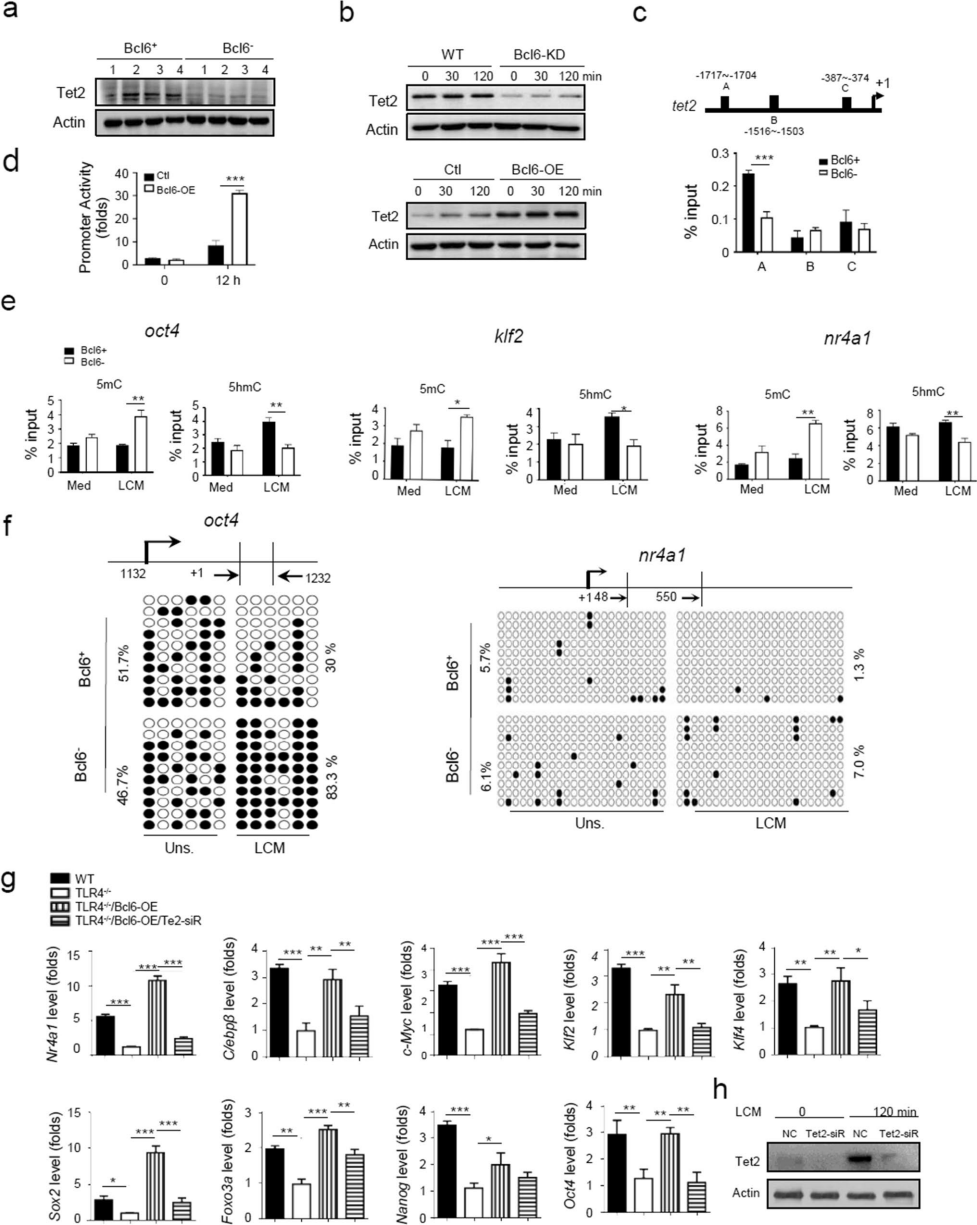
Bcl6 co-opts the demethylase Tet2 to shape SMMs epigenetic signature. **a** Immunoblotting of Tet2 in Bcl6^+^ and Bcl6^−^ TAMs isolated from tumors. **b** Immunoblotting of Tet2 either in WT or Bcl6-KD macrophages, or in TLR4^−/−^ macrophages transfected with Bcl6-expressing (Bcl6-OE) or control plasmids, followed by LCM stimulation for the indicated time periods. **c** The predicted Bcl6 binding sites at Tet2 promoter regions (upper); ChIP analysis of the Bcl6 enrichment at the *tet2* locus in Bcl6^+^ or Bcl6^−^ macrophages (lower). **d** Tet2-dependent promoter activity detected in RAW264.7 cells that were co-transfected with pGL3-Tet2 plasmids and Bcl6-OE or control plasmids, followed by LCM stimulation for 12 h.** e** CHIP analysis of 5mc and 5hmc levels at the genomic regions of representative lineage- or stem-related genes in Bcl6^+^ or Bcl6^−^ macrophages with or without LCM stimulation.** f** OxBS sequencing of methylated CpGs at the genomic regions of representative lineage- or stemness-related genes in Bcl6^+^ or Bcl6^−^ macrophages with or without LCM stimulation. Dots indicate methylated CpGs, and the percentages of methylated CpGs are shown.** g** qPCR analysis of the expression of lineage- and stem-associated genes, either in WT and TLR4^−/−^ BMDMs, or in TLR4^−/−^ BMDMs transfected with Bcl6-expressing plasmids (Bcl6-OE) alone, or co-transfected with Bcl6-OE and Tet2 siRNA. **h** Immunoblotting of Tet2 in BMDMs transfected with Tet2 siRNA or non-specific control (NC), followed by LCM stimulation for 2 h. Shown are representative images and the data from three independent experiments are expressed as means ± SEM. *p < 0.05, **p < 0.01, ***p < 0.001 by student’s *t* test

Trained immunity is typically recognized as long-lasting adaptation of innate immunity, which involves transcriptional and epigenetic modifications of myeloid cells and their bone marrow progenitors [26, 36–39]. As macrophage-specific enhancers have been reported to be pre-marked by DNA hydroxyl-methylation [28], we hypothesized that methylcytosine dioxygenase 2 (Tet2), a DNA demethylase critically involved in myelopoiesis and other immune cells differentiation [40], might participate in Bcl6-driven SMM differentiation program. Supportively, our data demonstrated that Tet2 was remarkably induced upon Bcl6 expression and reduced by Bcl6 deletion in tumor-conditioned macrophages (Fig. 6a, b). We further identified 3 putative Bcl6-binding sites within 5`UTR of *tet2* gene, among which site A (-1717 ~ -1704) was confirmed to be specifically tethered by Bcl6 (Fig. 6c). The dual luciferase assay also revealed that Bcl6 specifically induced Tet2-driven promoter activity (Fig. 6d). The data thus consistently supported the direct regulation of Tet2 expression by Bcl6.

Tet2 is known as a dioxygenase catalyzing the oxidation of 5-methylcytosine (5mC) to 5-hydroxymethylcytosine (5hmC), an active DNA demethylation permitting genes expression [40]. To examine the implication of the Bcl6/Tet2 in the epigenetic regulation of SMM-related genes, we then analyzed the enrichment of 5mC/5hmC at the loci of SMM-associated genes. The result showed that the residency of 5hmC at the loci of stem- and lineage-related genes, such as *oct4, klf2* and *nr4a1,* was increased in Bcl6-expressing macrophages, and the 5mC enrichment was decreased. On the contrary, Bcl6-silenced cells displayed decreased 5hmc/5mc ratio at the corresponding genomic loci (Fig. 6e), indicating that Bcl6 through Tet2 modulated the chromatin accessibility of stem and lineage-related genes. In support of this, the methylation of CpG islands at the *oct4* and *nr4a1* loci was decreased in Bcl6 sufficient macrophages but enhanced in Bcl6 depleting cells (Fig. 6f). The results thus indicated that SMM-related genes, which were likely “locked” by epigenetic marks in normal condition [28], was de-repressed by the demethylase Tet2 upon Bcl6 action in SMMs. Accordingly, deletion of Tet2 failed to fully activate SMM-related genes in tumor-conditioned macrophages even with Bcl6 overexpression (Fig. 6g, h). Collectively, our data revealed a Bcl6-driven and Tet2-mediated regulatory circuitry that dynamically controlled SMM-specifying program during tumor development.

### The AKT/mTOR pathway acts downstream of TLR4 to transnationally regulate Bcl6

**Fig. 7:**
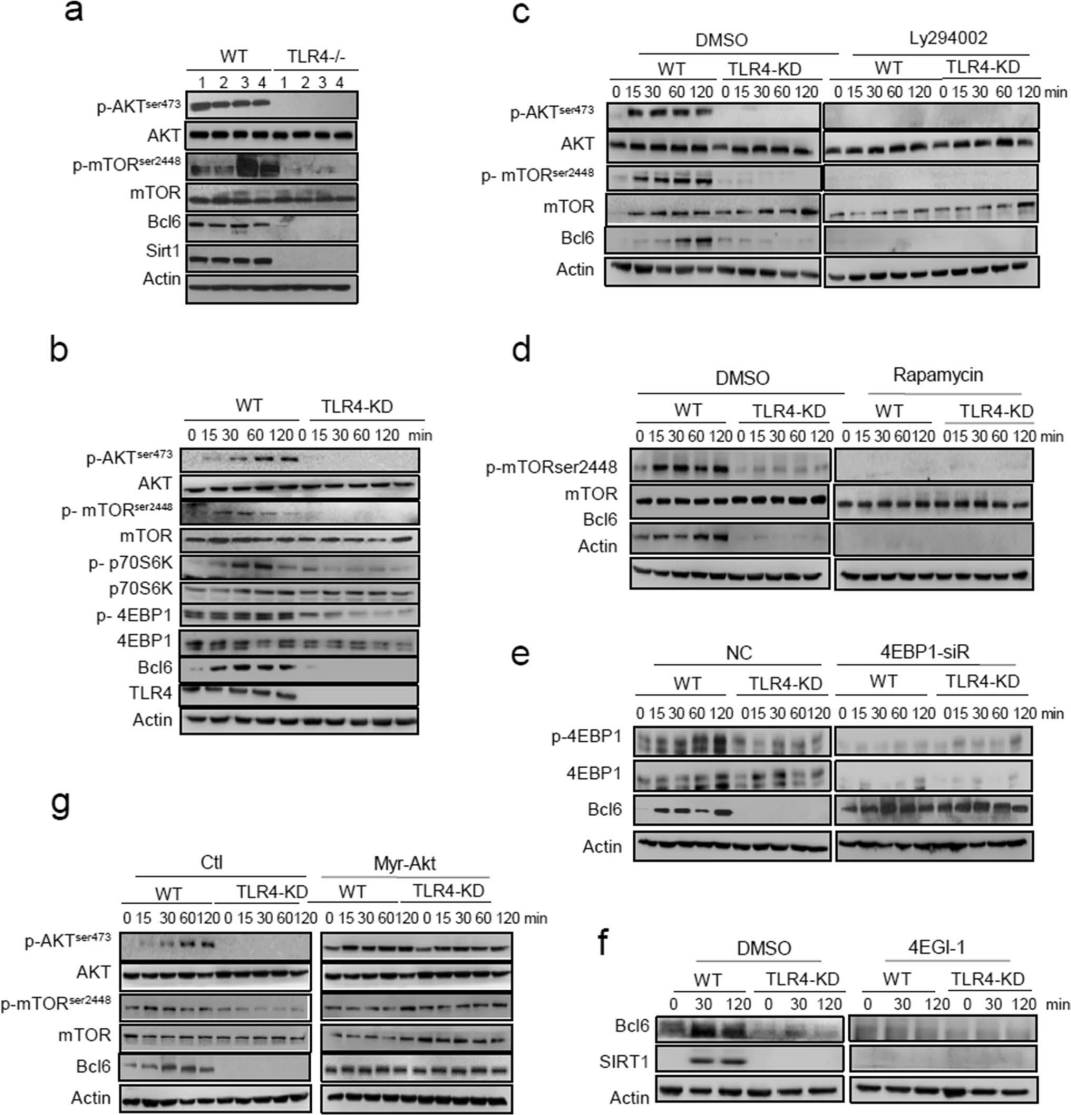
The AKT/mTOR pathway acts downstream of TLR4 to transnationally regulate Bcl6. **a** Immunoblotting of the indicated signaling molecules in TAMs isolated from WT and TLR4^−/−^ mice. The number 1–4 represent individual mice in distinct groups. **b** Immunoblotting of the indicated signaling molecules in WT and TLR4-knockdown (TLR4-KD) macrophages stimulated by LCM for the indicated time periods. **c**, **d** Immunoblotting of the indicated signaling molecules in WT and TLR4-KD macrophages that were pre-treated with DMSO or Ly294002, or with DMSO or Rapamycin respectively, followed by LCM stimulation for the indicated time periods. **e** Immunoblotting of the indicated signaling molecules in WT and TLR4-KD macrophages transfected with 4E-BP1-siRNA or non-specific control nucleotides (NC), followed by LCM stimulation for the indicated time periods. **f** Immunoblotting of Bcl6 and SIRT1 in WT and TLR4-KD macrophages that were pre-treated with DMSO or 4EGI-1(eIF4E inhibitor), followed by LCM stimulation for the indicated time periods. **g** Immunoblotting of the indicated signaling molecules in WT and TLR4-KD macrophages that were transfected with control (Ctl) or the constitutively active Akt (Myr-Akt) plasmids, followed by LCM stimulation for the indicated time periods. The representative images from 2–3 independent experiments were shown

As our initial study revealed that Bcl6 promoted SMMs differentiation via TLR4-initiated pathway, we next sought to address how tumor-derived signals induced TLR4 and subsequent Bcl6 pathway. Our attention was concentrated on mammalian target of rapamycin (mTOR), the serine/threonine kinase known for its ability to translate extracellular cues into intracellular signaling for cell fate decision. Indeed, our data showed that mTORC1 and AKT were activated, accompanied by the induction of Bcl6 and SIRT1in TAMs isolated from WT but not TLR4^−/−^ mice (Fig. 7a). The in vitro system also confirmed the AKT/mTOR and the downstream p70S6k and 4EBP1 was activated by LCM-treated WT but not TLR4^−/−^macrophages (Fig. 7b). By application of PI3K inhibitor Ly294002 or mTORC1 inhibitor rapamycin, we showed that Bcl6 induction was almost completely abrogated, further supported the dependency of PI3K/mTORC pathway on Bcl6 induction (Fig. 7c, d; Fig. S7a, b). mTORC1 is known as a key regulator of protein synthesis primarily through phosphorylation and de-repression of 4EBP1 action [41]. In accordance, our data showed that knockdown of the translational repressor 4EBP1 resumed the expression of Bcl6, alleviating its dependence of TLR4 signaling. Conversely, application of the Cap-translational inhibitor 4EGI-1 almost completely abolished Bcl6 expression induced by tumors, regardless of intact TLR4-sensing machinery (Fig. 7e, f). The results thus underscored the importance of mTOR-mediated protein synthesis pathway in the induction of Bcl6 in tumor-conditioned macrophages.

We next investigated the functional relevance of the PI3K/AKT/mTOR in SMM differentiation program. For this, a series of inhibitors targeting PI3K isoforms (α, β and γ) were applied to inhibit the pathway respectively to assess their effects on SMM-related gene expression (data not shown). Among these antagonists, PI3Kγ-selective inhibitor, isoindolinones, remarkably blocked the induction of stem-related genes in LCM-treated macrophages (Fig.S7c). We further used the myr-AKT plasmids to show that constitutive activation of AKT largely restored mTOR activation and subsequent Bcl6 expression even with no tumor instruction (Fig. 7g, Fig.S7d). Together, our data consistently demonstrated the PI3K/AKT/mTOR pathway played a pivotal role in translating tumor-derived signals into Bcl6-mediated SMMs differentiation program.

### Tumor-derived reduced HMGB1 serves as the priming signal for trained program in macrophages

**Fig. 8:**
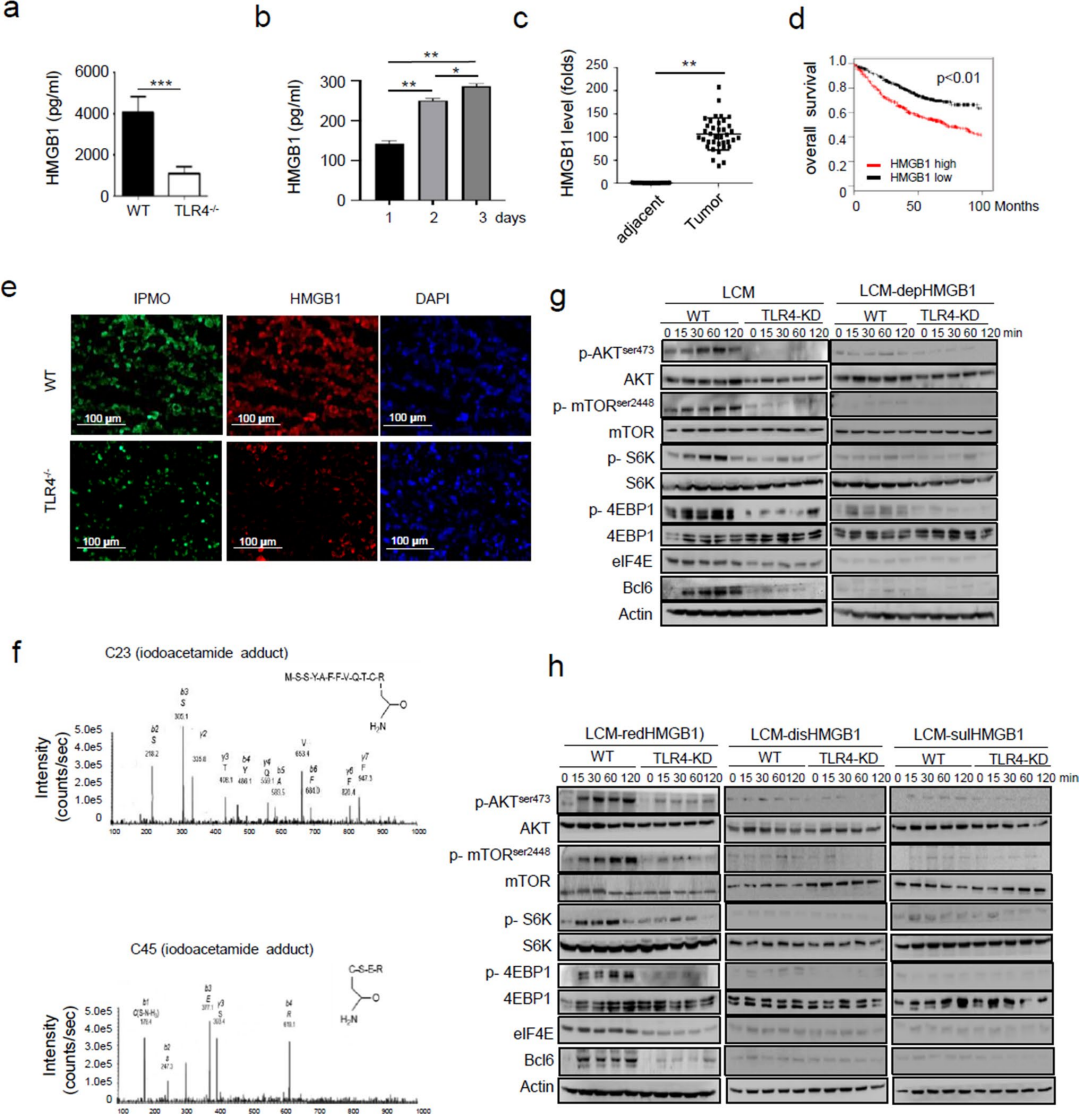
Tumor-derived redHMGB1 primes trained immunity in macrophages. **a** ELISA assay of serum HMGB1 levels in WT and TLR4^−/−^ mice 4 weeks post LLC implantation. **b** ELISA assay of supernatant HMGB1 in LLC cell culture at the indicated days. **c** qPCR analysis of HMGB1 levels in adjacent normal and tumor tissues of lung adenocarcinoma (AD) samples. **d** TCGA-based analysis showing the association of HMGB1 levels with overall survival rate of patients with lung AD. **e** Immunofluorescent staining of pimonidazole (PIMO, Green) and HMGB1 (Red) in tumor sections from WT and TLR4^−/−^ mice. **f** Mass spectrometry of serum HMGB1 from tumor-bearing mice with an iodoacetamide adduct indicating reduced C23 and C45. C23: MS–MS trace of the peptide containing amino acids 13–24; C45: MS–MS trace of the peptide containing amino acids 45–48. **g**, **h** Immunoblotting of the indicated signaling molecules in WT and TLR4-KD macrophages stimulated by LCM or HMGB1-depleted LCM (**g**), and HMGB1-depleted LCM with the addition of reduced HMGB1, disulfide HMGB1, or oxidizable HMGB1 respectively (**h**). Shown are representative images and the data from three independent experiments are expressed as means ± SEM. *p < 0.05, **p < 0.01, ***p < 0.001 by student’s *t* test

The question then remained unsolved was what kind of signal derived from tumors acted through TLR4 and mTORC1pathway to induce Bcl6-diriven program in TAMs. Among a wide spectrum of TLR4 ligands, high-mobility group box 1 (HMGB1) was known for its prominent role in regulating both immune responses and cancer progression. HMGB1 is a redox-sensitive signaling molecule generally released from stressed or damaged cells with the potential to induce innate immune responses and remold tissue microenvironment [42, 43]. Indeed, we initially observed that serum levels of HMGB1 in tumor-bearing mice, as well as that in supernatants of LLCs culture, were significantly increased (Fig. 8a,b). The qPCR analysis consistently demonstrated that HMGB1 was abundantly expressed at tumor tissues but not adjacent areas in samples of lung cancers (Fig. 8c). Also, TCGA-based analysis revealed an inverse correlation between HMGB1 level and outcome of lung cancer (Fig. 8d). The data thus supported the unfavorable role of HMGB1 in tumorigenesis. As studies have shown the redox state was critical for action of HMGB1 and the signaling pathway it induced [44], we therefore proceeded to determine its oxidize/redox form in tumor niches. By staining of tumor tissues with pimonidazole (PIMO), a widely used marker of cellular hypoxia, we demonstrated that tumor niches at 4 weeks post LLC inoculation were highly hypoxic (Fig. 8e). Accordingly, the mass spectrometric analysis revealed that HMGB1 accumulated in tumors were fully reduced, as Cys23 and Cys45 residues were conjugated with iodoacetamide, an agent specifically reacting with reduced thiols (Fig. 8f). The data thus confirmed the existence of reduced HMGB1 (redHMGB1) in ever-growing and hypoxic tumors.

We next explored the cellular source of redHMGB1 and the differential role of reduced or oxidized HMGB1 in the initiation of macrophage differentiation program. For this, we firstly prepared LCM harboring HMGB1 with differential redox forms. Cellular culture medium was collected from HMGB1-depleted LLC cells (HMGB1-dep), which was then complemented respectively with three forms of HMGB1, namely, reduced (redHMBG1), partially oxidized disulfide HMGB1 (disHMGB1), or fully oxidized sulfonyl-HMGB1 (sulHMGB1). Murine macrophages were subsequently cultured in these differentially prepared mediums. Impressively, the result showed that HMGB1-depleted medium failed to induce the AKT/mTOR pathway and subsequently Bcl6 induction, regardless of intact TLR4 on macrophages (Fig. 8g). Among three forms of HMGB1, only redHMGB1-repleted medium was able to induce mTOR activation and Bcl6 induction, as LCM normally prepared (Fig. 8h). The effect however could not be reproduced in TLR4 null macrophages, corroborating that tumor-derived redHMGB1, via TLR4-sensing pathway, initiated the mTOR/Bcl6 program in macrophages. More strikingly, we showed that adoptive transfer of macrophages that were pre-conditioned with HMGB1-depleted LCM yielded much smaller tumors than those pre-treated with HMGB1-sufficient LCM (Fig. S8a, b), highlighting the significance of tumor-derived HMGB1 in promoting the generation of pro-tumor macrophages. Together, the results identified tumor-derived redHMGB1 as a priming signal to drive TLR4/mTOR/Bcl6-mediated trained immunity in TAMs.

## Discussion

Compared with great advance achieved in our understanding about adaptive immunological memory, current knowledge of innate immunological memory is relatively rudimentary and mostly derived from the infection setting. The questions regarding specific signals and mechanisms underlying tumor-induced trained immunity remain largely unknown. By exploiting murine ectopic and orthotopic lung cancer models, we demonstrate that intratumoral macrophages undergo cellular differentiation and give rise to long-lived memory-like macrophages. Specifically, we identify a Bcl6^+^CD11b^+^F4/80^+^Ly6C^−^ macrophage subpopulation, which are featured with expression of stem-related genes, self-renewing potential, and importantly, long-term maintenance of pro-tumor activity, and therefore termed stem-like memory macrophages (SMMs). Furthermore, we identify Bcl6 as an obligate factor that drives SMM differentiation program while excluding alternative cell fate choice. In particular, Bcl6 co-opts the epigenetic modifiers Tet2 and SIRT1 to orchestrate stem-related gene program and mitochondria-biased metabolism, and hence bolster longevity and stability of trained program in TAMs. Additionally, our data reveal that tumor-derived redHMGB1 serves as the priming signal to be sensed by TLR4 and initiate the mTOR/Bcl6-driven program for SMMs development (Figures S8C). Together, we for the first time, to our knowledge, identify a distinct population of macrophages with a hybrid of stem and memory cell properties, and unveil a regulatory paradigm that integrates genetic, epigenetic and metabolic pathways to promote the generation of trained immunity for cancer development. The findings are of particular importance since we currently know little about innate immune memory induced by tumors, and provide new strategy for cancer immunotherapy by targeting memory-like macrophages.

Due to the intimacy between stem-related properties and memory-associated requirements, the concept of stem-like memory cells has been well described in T and B cells but not in innate immune cells ever [16, 17, 45]. Our present study demonstrates that Bcl6^+^ TAMs and their sibling cells stably maintain stem-related gene signature, self-renewing property and pro-tumor memory, even after culturing for an extended period in the absence of tumor stimulation. The findings imply that a hybrid status of stem and memory cells might be a general mechanism in both adaptive and innate immunological memory cells [46]. Since peripheral macrophages are generally considered differentiated cells with limited replicative potential, the adoption of stem cell-like property may have particular implication for the generation of long-lived innate immunological memory. Supportively, a recent study published in *Cell* revealed that there emerges a shared onco-fetal ecosystem during hepatocellular carcinoma (HCC) development leading to the re-emergence of fetal-like TAMs [47]. The study, in line with our data, supports that the de-differentiation process takes effect in TAMs to support long-lived trained immunity [17]. We further confirm that macrophages, either from bone marrows or tissue residency, can be instructed by tumor-derived signals to assume phenotypic and functional transition. This is congruent with previous report that TAM compartment is intermingled with both yolk sac–derived and monocyte-derived macrophages, and both of them would be substantially remolded and eventually convert into tumor-favoring phenotype, though the underlying mechanisms need to be further explored [48, 49].

In this regard, the transcriptional factor Bcl6 has been identified as a central factor in the present study to drive the differentiation program of SMMs. Bcl6 not only directly activates stem-associated genes that are otherwise silenced in mature macrophages, but also combines with the DNA methylate Tet2 to remold the epigenetic marks of lineage and stem-related genes, thereby facilitating the re-activation of primitive genes for generation of “fetal-like macrophages” [47]. Because the epigenetic factors like Tet2 generally lack specific gene binding domains, Bcl6 tends to not only regulate Tet2 transcription, but also directs Tet2 tethering to the loci of target genes [50]. As such, the coupling of transcriptional and epigenetic factors elaborates SMM-related gene program instructed by tumors [51].

In addition to acquiring stem-like properties, SMMs also require sufficient bioenergetics to support their long-term persistence and propagation, which is of particular importance in metabolically stressed TMEs. For this purpose, Bcl6 mediates a coherent metabolic program characterized by boosted mitochondrial respiration and restrained glycolytic catabolism. Through the SIRT1/PGC-1α axis, Bcl6 remarkably improves mitochondrial biogenesis, oxidative phosphorylation (OXPHOS) and ATP generation to support metabolic fitness of SMMs. Of interest, reinforced mitochondrial metabolism would additionally produce larger quantities of metabolic intermediates such as NAD^+^ and α-ketoglutarate (α-KG), which would serve as the substrates for SIRT1 and Tet2 to further promote SMM-specifying gene program [52]. As such, Bcl6-mediated metabolic rewiring not only enhances metabolic adaptiveness, but also promotes metabolic-epigenetic regulation for SMM development. Since development of distinct subset of immune cells is frequently coupled with shutdown of alternative cell differentiation program, it is expected to see the inhibitory role for Bcl6 in glycolytic pathway in TAMs. Loss of Bcl6 accordingly skews macrophages to glycolytic catabolism and pro-inflammatory phenotype, which tends to cause metabolic competition between TAMs and tumor cells, placing macrophages at proliferation and survival disadvantage in expanding tumors. This may provide the rationale for the shrinkage and even exhaustion of Bcl6^−^ macrophages in ever-growing tumors as we observed [25]. Thus, it is tentatively proposed that Bcl6 functions as an obligatory lineage transcription factor, as well as a metabolic checkpoint to guarantee long-lived memory-like macrophages generation while excluding alternative subsets differentiation.

Given the center importance of Bcl6 in governing SMMs development, there arises the question how Bcl6 is induced and sustains during tumor development. In the study, we reveal that tumor-derived signal, redHMGB1 specifically, is sensed by TLR4 on TAMs and acts through mTORC1-mediated translational machinery to control Bcl6 expression. The finding thus links mTORC1, a well-established regulator for trained immunity during infection [5, 9, 22, 41] with tumor-induced memory-like macrophage development. Recent data indicate that mTORC1-mediated translational machinery is essential for nucleus-encoded mitochondrial genes expression and hence mitochondrial activity [53]. Our data demonstrate that mTORC1 may also act through Bcl6 to boost mitochondrial biogenesis and function, thus connecting metabolic pathway with Bcl6-driven lineage gene program. Additionally, our data indicate that Bcl6 is rapidly induced upon tumor stimulation, implying that there might exist pre-synthesized Bcl6 mRNA, facilitating its rapid elicitation upon stimulation and setting the basis for recall-like responses. Indeed, early induction of Bcl6 is critical for SMM commitment and memory formation during cancer development, as our data demonstrate that interference of Bcl6 signaling at the priming stage substantially affects the differentiation of memory macrophages, whereas blocking of Bcl6 signaling at later period only yields mild effect on tumor eradication. Additionally, Bcl6 itself has been modified by methylation [54], and Tet2 induction would remove the repressive moieties from Bcl6 loci and reinforce its expression. This leads to the speculation that Bcl6 cooperates with Tet2 to form a positive feedback circuitry, ensuring rapid and efficient formation of trained innate immunity.

Finally, we identify tumor-derived redHMGB1 as the priming signal driving trained program in TAMs. HMGB1 is a conical danger signal released from stressed or damaged cells, and its relevance to tumor progression has been documented [55]. Our data indicate that, besides the expressive level, the oxidative/redox status of HMGB1 potentially impinges its biological activity. The priming effect of redHMGB1 appears not due to the chemoattractive activity, since application of non-oxidizable HMGB1, the factor presumed to have no other activity but chemoattractance [44], exhibits insignificant impact on Bcl6 induction. Although the regulatory mode exploited by HMGB1 with different redox forms remains elusive at present stage, emerging evidences have suggested that the oxidative/reductive modification may substantially alter the molecular configure and hence the ligation of HMGB1 with its cognate receptors, thereby affecting the downstream signaling [42, 56]. TLR4 is a best-characterized PRR capable of recognizing multiple ligands such as LPS, HMGB1, S100A9 and hyaluronan, and mediating discrete intracellular pathway [34, 57, 58]. Studies have revealed that the receptor signal strength is critical for immune memory formation, with stronger signals favoring short-lived effector cells and weaker signals fostering long-lived memory cells [59]. Future studies might be merited to quantitatively gauge the receptor signaling formed by different TLR4 ligands, particularly HMGB1 with different redox modification, which is believed to give more mechanistic insight into context and signal-specific trained innate immunity.

In summary, our study unveils a distinct macrophages subpopulation that was driven by Bcl6 to persistently promote cancer progression. Further studies might be merited to further characterize the stemness and memory-like properties associated with intratumoral macrophages.

## Materials and methods

Patients’ lung cancer tissues were obtained from Zhejiang Cancer Hospital and the affiliated Hospital of Hangzhou Normal University, and the patients gave written informed consent to participate in the study that took place at Zhejiang Cancer Hospital and the affiliated Hospital of Hangzhou Normal University. Patient inclusion procedures, sample acquisition and preparation, data handling were performed according to the Declaration of Helsinki. The research protocol was approved by the institutional Review Board of affiliated Hospital of Hangzhou Normal University. The entire experimental protocol was conducted in compliance with the institutional guidelines.

### Mice

TLR4^−/−^ and littermate wild-type controls (WT) mice were kindly provided by Dr. S. Akira (Osaka University, Osaka, Japan) and maintained in a pathogen-free rodent barrier facility. All of these mice were backcrossed for at least eight generations onto C57BL/6 background. They were housed in a controlled environment and provided with standard rodent chow and water. Animal experiments were performed according to the NIH Guide for the Care and Use of Laboratory Animals, with the approval of the Scientific Investigation Board of Nanjing University of Traditional Chinese Medicine (No. 202109A023). All mice in our experiments were gender and age matched.

### Lung cancer generation

For the generation of lung cancer model, 5 × 10^5^ Lewis lung cancer cells (LLCs) were injected subcutaneously (s.c) into the right side of the rear flank of mice. Tumor size was assessed one per week by a digital caliper. The tumor volumes were determined by measuring the length (l) and the width (w) and calculating the volume (V = lw^2^/2). Mice were sacrificed 2, 3, 4 weeks after injection.

For the generation of lung cancer inhibit model, 1 × 10^6^ Lewis lung cancer cells were mixed with 500 ug macrophage-targeting peptide embedding Bcl6 inhibitor RI-BPI or none (Pep), and then injected subcutaneously into the right side of the rear flank of mice. At 3–19 days post injection, the Pep or RI-BPI was injected into tail vein every two days. Mice were sacrificed 4 weeks after injection.

For generation of orthotopic lung cancer model, LLC cells (1 × 10^6^ in 50 ul) were suspended in 50 ul Matrigel and injected into the parenchyma of the left lung lobe through the rib cage using a 30-gauge needle as previously described [60]. During injection, the lung was visualized through a 4- to 5-mm incision in the skin. Mice were sacrificed 2 and 3 weeks after injection.

### Peritoneal macrophage isolation

Peritoneal macrophages were obtained in the abdominal cavity by inoculation of 3–5 ml of cold PBS. The peritoneal liquid was collected and put in centrifugal tubes for four times. The tubes were centrifuged at 200 × g for 5 min. The cells from each individual mouse were resuspended in cell culture medium and cultured in a *10-cm* diameter culture *dish*. The cells were incubated at 37 °C and, after 2 h, non-adherent cells were removed and the adherent cells were collected and used as peritoneal macrophages.

### Bone marrow-derived macrophages (BMDMs) isolation

For the generation of BMDMs, murine bone marrow cells were extracted from the leg bones and differentiated in DMEM (Gibco, ThermoFisher Scientific, Waltham, USA) containing 10% fetal calf serum, 1% penicillin streptomycin and 20 ng/ml M-CSF for 6 days. Non-adherent cells were removed and the adherent cells were collected. Next, BMDMs were purified by positive selection from adherent cells using the magnetic cell sorter (MACS) CD11b microbeads from Miltenyi Biotecthe.

### Tumor-associated macrophages (TAMs) isolation

Tumor tissue was cut into 2 mm sections and digested with type I collagenase (0.3 mg/ml) for 1 h at 37 °C. The suspension was filtered through a 70 μm stainless steel wire mesh to generate the single-cell suspension. The suspension was centrifuged and washed twice with PBS. Cells were then left to adhere in serum-free DMEM for 40 min. After that, non-adherent cells were washed away. The adherent cells were stained with CD45, Ly6G, F4/80 and CD11b for purifying the macrophages by flow cell sorter. Immunofluorescence staining for macrophage markers (CD45^+^Ly6G^−^F4/80^+^CD11b^+^) confirmed the identity of the adherent populations.

### LLCs isolation

To recover tumor cells from tumors as previously describe [61], tumor tissue was cut into 2 mm sections and digested with type I collagenase (0.3 mg/ml) for 1 h at 37 °C. The tissue debris was removed by centrifuging. After that, cells were left to adhere in serum-free DMEM for 40 min. Non-adherent cells were rinsed with DMEM containing 10% FBS, and then plated in culture plate. Cells were passed for 3 generations at 1:5 before harvested as LLCs. To assess the purity of tumor cells, TLR4 was detectable by immunofluorescent staining in tumor cells harvested from TLR4^−/−^ mice, indicating that there was no significant contamination by host cells.

### Macrophage depletion

For macrophages depletion, 200 μl of clodronate liposomes was intraperitoneally injected in mice the first day. On day 4, 100 μl of clodronate liposomes was intraperitoneally injected again; on day 4, 1 × 10^6^ LLCs were subcutaneously injected. On day 8, 12, 16, 20, 24, and 28, mice were injected into 100 μl of clodronate liposomes intraperitoneally injected every day. The mice were killed on day 32.

### Cell invasion assay

A Boyden chamber (BD Biosciences, San Jose, CA, USA) with a pore size of 8 μm was used for the in vitro cell invasion assay. Serum-starved cells (5 × 10^4^) were seeded in fibronectin-coated chambers, and fetal bovine serum-containing medium was used as chemoattractant in the bottom chamber. After 24 h of incubation at 37 °C, cells invading the lower surface of the membrane were stained with 1% Toluidine Blue, and quantified by fluorescence microscopy (Nikon E600, Nikon Instrument Inc., Tokyo, Japan).

### Cell migration assay

Tumor cells were seeded in p60 plates (Corning, Corning, NY, USA) and allowed to grow to full confluence. Then the monolayer of cells was scratched using a 1 ml pipette tip, and the migration distances were photographed at 0 and 24 h after scratching. Five fields were randomly selected for each experiment, and the migratory cells were counted using Metamorph software (Nashville, TN, USA).

### Cell proliferation assay

Cell proliferation was measured using the 3-(4,5-dimethylthiazol-2-yl-5-(3-carboxymethoxyphenyl)-2-(4-sulfophenyl)-2H-tetrazolium (MTS) assay according to the manufacturer’s protocol (Promega, Madison, WI, USA). Cells (1.5 × 10^3^) were seeded into 96-well plates, and 20 μl of 5 mg/ml MTS was added to each well. After 1 h of incubation, cell viability was assessed by measurement of absorbance at 490 nm using a microplate reader. For the cytotoxicity analysis, cells were exposed to gefitinib (0, 1, 2, 3, 4, 5, 6 μM) for 24 h and subjected to the MTS assay.

### Colony formation assay

For colony formation assay, tumor cells (1 × 10^4^/well) were resuspended in a medium containing 0.3% low-melting-point agarose and were plated on top of the bottom agar that consisted of growth medium containing 10% fetal bovine serum and 0.75% agarose in six-well plates. The cells were incubated at 37 °C in 5% CO2, and 20 days later colonies were stained with 1 mg/ml 3-(4,5-Dimethylthiazol-2-yl)-2,5-diphenyltetrazolium bromide (MTT) for 6 h. The numbers of colonies larger than 100 μm in diameter were counted.

### Spheroid-forming assay

The isolated macrophages or tumor cells were seeded into 24-well ultra-low adherent plates (Corning, USA) at 1000 cells/well in 1 ml SCM (Stem Cell Medium, Gibco, ThermoFisher Scientific, Waltham, MA, USA) for 7 days. The number of spheres with diameter ⩾70 μm was determined using an inverted fluorescence microscope (Nikon). Sphere formation efficiency (SFE) was calculated as the percentage of seeded cells that gave rise to a spheroid.

### Immunohistochemistry (IHC) staining

Sections of paraffin-embedded specimens were deparaffinized in xylene and rehydrated in graded alcohol washes. On antigen retrieval, the slides were incubated respectively with antibodies against mouse vimentin (VIM), MMP9, Ki-67, PCNA at room temperature for 1 h, followed by incubation of the secondary antibodies. Diaminobenzidine-hydrogen peroxide was the chromogen, and the counterstaining was carried out with 0.5% hematoxylin.

### SMMs culture

Macrophages isolated from tumors were seeded with DMEM into a 10 cm dish and incubated for 40 min. After washing away non-adherent cells, adherent cells were seeded into 24-well ultra-low adherent plates (Corning, USA) at 1000 cells/well in 1 ml SCM harboring 20 ng/ml M-CSF for 7 days. Then 1 ml DMEM containing 10% FBS and 1% penicillin streptomycin was added per 2 days for 5–6 weeks.

### Quantitative RT-PCR

Total RNA isolated was reverse transcribed using the Superscript III system (Invitrogen, Carlsbad, CA, USA). qPCR was carried out in triplicate with the SYBR Green method, and relative expression levels were determined by applying the ΔΔCt method using β-actin as the endogenous control. Primer sequences were used in Table S1.

### IHC quantification

The IHC images was quantified through Image J software. By using IHC Profile plug-in, a fractional ROI (Region of Interest) can be gated. Then pressed the training button and convert the image format to 8 bit so that all the ROI can be chosen. The selected ROI can be measured for its IOD (integrated option density) and area. Then calculated AOD by the equation: ∑ Integral optical density (IOD) / ∑ Area. The results were analyzed in GraphPad Prism software.

### Annexin V /PI experiment

For cell apoptosis assay, single-cells were resuspended by 100 μl precooled 1 × Annexin V Binding Buffer. Then 5 μl Annexin V-EGFP, 3 μl PI were added in and shook gently for mixing well. Cells were incubated at room temperature for 15 min. 400 μl precooled 1 × Annexin V Binding Buffer were added in and mixed gently. The stained cells were analyzed by flow cytometry.

### 5mC and 5hmC meDIP-qPCR

Methylated DNA immunoprecipitation (meDIP) was performed using the Active motif MeDIP or hMeDIP Kit with minor modifications. DNA was sonicated into short fragments with a Branson sonicator for 20 min with 15-s on, 15-s off cycles at low power. Sonicated DNA was heat denatured at 95 °C for 10 min. Sonicated DNA (1 μg) was immunoprecipitated with 1 μg of mouse anti-5-methylcytosine monoclonal antibody or 2.5 μg of mouse anti-5-hydroxymethylcytosine monoclonal antibody). After overnight incubation at 4 °C, magnetic beads were added to the DNA-antibody mixture and samples were incubated at 4 °C for 2 h. Isolation of immunoprecipitated DNA was performed according to the kit instructions. qPCR was performed using SYBR® Green Supermix (Bio-Rad) on a Bio-Rad CFX96 Real Time system, as indicated by the manufacturers protocol. The percent enrichment was calculated as compared with the amount of DNA used in the IP reaction. Primer sequences were used in Table S1.

### Chromatin immunoprecipitation (ChIP) assay

Cells were fixed, nuclei lysed, and chromatin sheared with a Branson sonicator. Immunoprecipitation was performed with the Millipore ChIP kit according to the manufacturer’s protocol. To precipitate DNA–protein complexes, anti-Bcl6 antibody was used and goat IgG was used as an isotype control. Percentage input was determined by removing an aliquot of sheared chromatin prior to immunoprecipitation and comparing amplification of this DNA with amplification of the precipitated chromatin. Putative Bcl6 binding sites in the *klf2, klf4, sox2, c-myc, oct4, sirt1, hif-1α, tet2* loci were investigated to assay the relative enrichment by qPCR. Primers were listed in Table S1.

### Immunoblotting and immunofluorescence

Cell lysates were prepared by lysing 2 × 10^6^ cells in lysis buffer (1% Triton X-100, 1% deoxycholate, 0.1% NaN_3_) containing protease inhibitor cocktail tablets (Roche Diagnostics,). Equal amounts of protein were separated on 10% SDS–polyacrylamide mini-gels and transferred to Immobilon PVDF membranes (Millipore, Boston, MA, USA). After blocking in Tris-buffered saline with Tween-20 (TBST) containing 5% bovine serum albumin, the membranes were incubated with the appropriate primary antibody, followed by a secondary antibody conjugated to horseradish peroxidase (Santa Cruz Biotechnology, Dallas, TX, USA). The signals were visualized with western blotting substrates.

For immunofluorescence detection, tumor tissue sections were fixed in 4% paraformaldehyde at room temperature for 5 min, washed with PBS twice, incubated with 1% BSA at 37 ◦C for 30 min to block nonspecific interactions, and then stained with primary antibodies to CD68 (8ug/ml), TLR4(5ug/ml), Bcl6 (2ug/ml), IPMO, and HMGB1(5ug/ml) for 1 h (Santa Cruz Biotechnology or R&D system). Sections were then incubated in rhodamine-labeled goat anti-mouse secondary antibody (Proteintech Group, Inc., Chicago, USA) at room temperature for 1 h. Nuclei were then stained with 4, 6-diamidino-2-phenylindole.

### Enzyme-linked immunosorbent assay

Cytokine or HMGB1concentrations in cell supernatants or serum samples were measured using ELISA. ELISA kits for mouse HMGB1 according to the manufacturer’s instructions (Biorbyt, UK). Optical density values were measured at a wavelength of 450 nm, using a FLUOstar Optima plate reader (BMG Labtech). Concentrations were calculated using a 4-parameter fit curve.

### Flow cytometry

Single-cell suspensions were stained and subsequently analyzed by a BeckmanCoulter gallios flow cytometer (BeckmanCoulter), and data were analyzed using FlowJo software. The antibodies used in the study including PE-Cy5-labelled anti-CD45, APC-Cy7-labelled anti- CD3, PE-Cy7-labelled anti-Ly6G, FITC-labelled anti-CD11b, PE-labelled anti-F4/80, PerCy5.5-labelled anti-Ly6C and APC-labelled anti-Bcl6 were purchased from Biolegend or eBioscience. The ALDEFLUOR assay (Stem Cell Technologies) was performed to identify cells with high aldehyde dehydrogenase (ALDH) activity according to the manufacturer’s protocol. For cell surface protein labeling, cells were incubated with anti-CD44-APC, anti-CD24-PE for 30 min at 37 ºC. For mitochondrial staining, cells were incubated with 50 nM MitoTracker Red CMXRos, Mito Treaker Green FM or 5 nM Mitosox for 30 min at 37 ºC in 5% CO2.

### Bcl6^+^ SMMs sorting

Single-cell suspensions prepared from tumors were stained and sorted by a BD FACSAria III flow cytometer. The antibodies used in the study including FITC-labelled anti-CD11b, PE-labelled anti-F4/80 and APC-labelled anti-Bcl6 were purchased from Biolegend or eBioscience. The Bcl6^+^ cells were picked up from CD11b^+^F4/80^+^ macrophages by flow cytometer. For cell labeling, cells were incubated with FITC-labelled anti-CD11b, PE-labelled anti-F4/80 for 30 min. After that, cells were washed, fixed and permeabilized with Cytofix/Cytoperm buffer for 40 min and then intracellularly stained with APC-labelled anti-Bcl6 for 30 min before cell sorting. Alternatively, in some experiments wherein live cells were necessary (e.g. proliferation, spheroid-forming assay, cellular metabolism), TAMs were sorted from TLR4^+/+^ and TLR4^−/−^ mice at 3–4 weeks post LLC inoculation, as the data consistently revealed that more than 95% of isolated TLR4^+/+^ TAMs were Bcl6 positive and above 98% of TLR4^−/−^ TAM were Bcl6 negative in this setting.

### siRNA and plasmid transfections

Cox15, Bcl6, SIRT1, HIF1-α, HMGB1, Tet2-specific siRNA and non-specific control (NC) siRNAs were purchased from Genepharma. Cells were seeded into 6-well plates at a density of 1 × 10^5^ cells/well in 2 ml complete medium. After overnight incubation, the media was removed and replaced with 500 μl serum-free media and the Lipofectamine RNAiMAX (Thermo Scientific, USA) /siRNA complexes were added to each well. After 4 h, the media was replaced with 2 ml fresh medium containing 10% FBS, and incubated for another 24 h or 48 h at 37 °C. All transfection experiments were performed in triplicate.

The constitutively active Akt or Tet2 plasmids or empty plasmids were purchased from Addgene. Bcl6- or SIRT1-expressing plasmids were purchased from GeneChem. Cells were seeded into 6-well plate the day before transfection. The myr-Akt1 or empty plasmids were transfected with Lipofectamine 2000 (Invitrogen, USA) according to the protocol suggested by the manufacture. After 48 h of transfection, the positive clones were selected.

### Plasmid construction and luciferase reporter assay

To directly detect the regulatory effect of Bcl6 on the targeted genes, the 5′ UTR of the genes were amplified from genomic DNA and cloned in PGL3 vector. The binding sequences-mutant plasmids were generated by PCR-based site-directed mutagenesis. The reporter plasmids and pEGFP-Bcl6 vector were then transfected into RAW264.7 cells using Jet-ENDO transfection reagents (Polyplus, Illkirch, France). 6 h and 24 h later, the cells were collected and assayed for firefly luciferase activities (Promega, USA) with or without LCM stimulation.

### Seahorse analysis of oxygen consumption and lactate production

Cells were plated at 0.5 × 10^4^ cells/well on a 96-well Seahorse plate, with one well per row containing only supplemented media as a negative control. A utility plate containing calibrant solution (200 μl/well), along with the plates containing the injector ports and probes was placed in a CO_2_-free incubator at 37 °C overnight. Media was then replaced by glucose-supplemented XF assay buffer (200 μl/well). Plates were then placed in a CO2-free incubator for at least 0.5 h. The inhibitors such as Oligomycin, carbonyl cyanide-4- (trifluoromethoxy) phenylhydrazone (FCCP), 2DG, inhibitor A + B were added to the appropriate port of the injector plate. The plates were run on the Seahorse for calibration.

### ATP measurement

Macrophages were plated at 5 × 10^5^ cells/ml in white 96-well plates (100 μl/well) and treated as required. ATP was assayed using an ATP quantification bioluminescent Kit (Abcam) according to the manufacturer’s instructions.

### Quantification of mitochondrial DNA

Mitochondrial DNA extracted from the cells was quantified using the Mitochondrial DNA (mtDNA) Monitoring Primer Set (Takara) according to manufacturer’s guidelines. Primer sets for ND1 and ND5 were used for the detection of mtDNA.

### Transmission electron microscopy

The isolated macrophages were fixed in 2% paraformaldehyde, 2.5% glutaraldehyde in 100 mM sodium cocodylate containing 0.05% malachite green. Samples were then washed in cocodylate buffer and fixed in 1% osmium tetroxide. After extensive washing in H_2_O, samples were stained with 1% aqueous uranyl acetate for 1 h and washed again, followed by dehydration in ethanol and embedding in Eponate 12 resin (Ted Pella). Sections were stained with uranyl acetate and lead citrate and imaged using a JOEL 1200 EX transmission electron microscope equipped with an 8 MP ATMP digital camera (Advanced Microscopy Techniques).

### Adoptive transfer

For the adoptive transfer assays, 2 × 10^6^ macrophages were i.v. injected into mice one week after inoculation of LLCs. Tumor size was measured each week. The mice were sacrificed 4 weeks, after adoptive transfer and tumor tissues were dissected out for further analysis.

### Mass-spectrometric characterization of the cysteine oxidation status of HMGB1

Reduced cysteine residues within HMGB1 from LLC cells in serum-free medium were characterized by thiol-specific alkylation with 50 mM iodoacetamide. Alkylation with iodoacetamide yields a mass-shift of 57 amu (atomic mass unit). HMGB1 was isolated by immunoprecipitation from LLC cells in serum-free medium as described previously [62]. HMGB1 preparations were then separated by non-reducing SDS PAGE and protein bands corresponding to the molecular weight of HMGB1 were excised. Recovered protein was then subjected to tryptic digestion and the resultant peptides were characterized by liquid chromatography–electrospray tandem mass spectrometry (LC–MS/MS) as described previously. Individual peptide fragmentation to produce *b* and γ ions was used to determine the amino acid sequence and confirm the presence of specific modifications.

### Brdu tracing and immunodetection

According to previous study [63], 1 × 10^5^ BMDMs were cultured in 6- well plate for 48 h, then incubated with 20 umol/L BrdU for 48 h. 2 × 10^6^ BMDMs were injected into the tail veins of mice one week after inoculation of LLCs. For the detection, single-cell suspensions obtained from tumor were stained with PE-labelled anti- Brdu.

### Statistical analysis

Graphs were made and statistical analysis was performed using Prism software (Graphpad). All quantitative data are presented as mean ± SEM. The criterion for significance was set at *p* < 0.05. Correlations between two parameters were assessed by Pearson’s correlation analysis. The cumulative survival time was calculated using the Kaplan–Meier method. The data were analyzed using two-tailed tests, and *p* < 0.05 was considered the standard of statistical significance. *p < 0.05, **p < 0.01, ***p < 0.001.

### Supplementary Information

Below is the link to the electronic supplementary material.

Supplementary file1 (DOCX 3911 KB)

## Data Availability

All data and information regarding material will be available upon reasonable request.
