# Supplementary material for: Bcl6 drives stem-like memory macrophages differentiation to foster tumor progression
Source: Cell Mol Life Sci. 2022 Dec 21;80(1):14. doi: 10.1007/s00018-022-04660-0 (PMC9771855; doi:10.1007/s00018-022-04660-0)
Supplement: Supplementary file 1 — Supplementary file1 (DOCX 3911 KB) [file 18_2022_4660_MOESM1_ESM.docx]

**Figure S1. TLR4 on macrophages is critical for cancer progression.** WT and TLR4^-/-^ mice (n=3) were s.c. injected with LLCs (5×10^5^/mice). 4 weeks later, mice were sacrificed and singe cell suspension (SCS) was prepared. **(a)** Quantitative analysis of the indicated malignancy-associated molecules in tumor tissues. **(b)** The survival rate of tumor cells upon gefitinib treatment at the doses indicated. **(c)** Flow cytometry of the frequency of CD3^+^CD8^+^ CTLs in tumors. **(d)** Intracellular staining and flow cytometry of the IFN-γ-producing CTLs (CD3^+^CD8^+^). **(e)** Intracellular staining and flow cytometry of Foxp3^+^ Treg (CD4^+^CD25^+^). **(f)** Flow cytometry of the depletion efficacy of TAMs upon chlodronate treatment. The frequency of F4/80^+^CD11b^+^ intratumoral macrophages were analyzed mice receiving chlodronate three times one week. **（g）**A representative image from qPCR assay of TLR4 and CD68 levels in cancerous or adjacent tissues from NSCLC patients are shown. Shown are representative images and the data from three independent experiments are expressed as means ± SEM. *p < 0.05, **p < 0.01 by student’s *t* test.

**Figure S2. The generation of Bcl6^+^ SMMs is instructed by tumor-derived signals. (a)** Flow cytometry of single cell suspension of tumors from WT and TLR4^-/-^ mice at 2-, 3- and 4-weeks post LLCs implantation. Intratumoral macrophages were gated on CD45^+^CD3^-^Ly6G^-^F4/80^+^CD11b^+^. **(b)**Flow cytometry of purity of tumor-associated macrophages (CD45^+^Ly6G^-^F4/80^+^CD11b^+^) sorted by BD FACSAria™ III sorter. **(c)**The percent of Bcl6^+^ SMMs (Ly6C^-^CD11b^+^F4/80^+^) developed from BMDMs cultured in fresh medium alone (ctl) or LLC-conditioned medium (LCM) for 24 h. **(d)**Flow cytometry of the percentage of Bcl6^+^ SMMs (Ly6C^-^CD11b^+^F4/80^+^) developed from peritoneal macrophages conditioned by LCM. **(e)**Immunoblotting of Bcl6 in TAMs isolated from WT or TLR4^-/-^ mice (upper), or in WT and TLR4-knockdown (TLR4-KD) macrophages stimulated by LCM for the indicated time periods. Shown are representative images and the data from three independent experiments are expressed as means ± SEM. *p < 0.05, **p < 0.01, ***p < 0.001 by student’s *t* test.

**Figure S3. Bcl6^+^ SMMs are consistently generated in a murine orthotropic model.** LLCs (1×10^6^/mice) were injected into lungs of WT and TLR4^-/-^ mice (n=3). 2 or 3 weeks later, mice were sacrificed and tumors were collected. **(a)**The weights of lungs in WT and TLR4^-/-^ mice at 2, 3 weeks. **(b)** H&E staining of lung tissues. **(c)**Flow cytometry of Bcl6^+^ macrophages in tumor cell preparation. The amounts of Bcl6^+^ SMMs were analyzed by gating at CD3^-^Ly6G^-^ F4/80^+^CD11b^+^ and further stratified by Ly6C and Bcl6 levels. The data are expressed as means ± SEM. ***p < 0.001 by student’s *t* test.

**Figure S4. Bcl6^+^ SMMs retain phenotypic and functional properties initially formed in tumor niches. (a,b)**Flow cytometry of macrophage-related surface markers **(a)**, and Heatmapping of immune-related molecules **(b)** in Bcl6^+^ or Bcl6^-^ SMMs sorted from single cell preparation of tumors. **(c,d)** LPS-induced endotoxic mice (n=12) were adoptively transplanted with spheroid-generating macrophages that were in vitro cultured for 5 weeks. H&E staining of lung tissues **(c)** and Kaplan-Meier survival analysis of the endotoxic mice **(d)**. (**e-h**)Bcl6^+^ TAMs were stimulated with LPS (100ng/ml)/IFNɣ (20ng/ml) for 48 h. Heatmapping of immune-tolerant markers (**e**); qPCR analysis of stem-related genes (**f**); Spheroid-forming assay of cellular self-renewing capability (**g**); Heatmapping of pro-inflammatory cytokines (**h**). p < 0.05 by log-rank test. Shown are representative images. The data from three independent experiments are expressed as means ± SEM.

**Figure S5.** **Bcl6 regulates metabolic programs of SMMs. (a,b)** ATP generation and Lactate production in BMDMs transfected with Bcl6-specific or non-specific siRNA, followed by LCM stimulation for the indicated time periods. **(c)**The volume and weight of tumors developed in Bcl6^fl/fl^ and Bcl6^∆LysM^ mice transplanted with LLCs. **(d)** Flow cytometry of macrophages in tumors developed in Bcl6^fl/fl^ and Bcl6^∆LysM^ mice at the indicated time periods post LLC inoculation. Live cells were gated on CD45^+^CD3^-^Ly6G^-^F4/80^+^CD11b^+^ for macrophages and further stratified by Ly6C and Bcl6 level. **(e-h)** Bcl6 ^fl/fl^ and Bcl6^∆LysM^ macrophages were sorted from tumor-bearing mice at 24 days post LLC inoculation and subjected to functional analysis. Spheroid-forming assay of cellular self-renewing capability (**e**); qPCR analysis of stem-related genes (**f**); ATP generation (**g**); Amounts of dysfunctional mitochondria by staining with MitoTracker Red and MitoTracker green (**h**). **(i,j)**ATP generation and Lactate production in BMDMs transfected with Bcl6-expressing (Bcl6-OE) or control (Ctl) plasmids, followed by LCM stimulation for the indicated time periods. **(k)**The immunofluorescent staining of F4/80 and CSF-1R in murine tumors injected with RI-BPI-harboring or control nanoparticles. Shown are representative images. The data from three independent experiments are expressed as means ± SEM. **p < 0.01, ***p < 0.001 by student’s *t* test.

**Figure S6. Bcl6 acts through SIRT1 to modulate mitochondrial activity in SMMs. (a,b)**Mito-Green/Red staining and ATP generation in BMDMs that were transfected with SIRT1-specific siRNA or non-specific (NC) nucleotides, followed by LCM stimulation for the indicated time periods. **(c,d)** Mito-Green/Red staining and ATP generation in BMDMs that were transfected with SIRT1-expressing (SIRT1-OE) or control (Ctl) plasmids, followed by LCM stimulation for the indicated time periods. The data from three independent experiments are expressed as means ± SEM. *p < 0.05, **p < 0.01, ***p < 0.001 by student’s *t* test.

**Figure S7. The AKT/mTOR pathway is essential for tumor-elicited Bcl6 expression and SMM-related gene program. (a,b)** Heatmapping of Bcl6 and stemness-associated molecules in WT or TLR4-KD macrophages that were treated respectively with DMSO/Ly294002 (**a**), DMSO/Rapamycin (**b**) followed by LCM stimulation for the indicated time periods. **(c)** Immunoblotting of the indicated molecules in WT and TLR4-KD macrophages that were pre-treated with DMSO or PI3Kγ inhibitor. **(d)** Heatmapping of Bcl6 and stemness-associated molecules in WT or TLR4-KD macrophages that were treated with Myr-Akt or control plasmids followed by LCM stimulation for 2 h. Shown are representative images of similar results from three experiments.

**Figure S8. Depletion of tumoral HMGB1 abrogates pro-tumor activity of macrophages. (a,b)** Size and weight of tumors developed in mice (n=4) that were adoptively transplanted with macrophages pre-conditioned with control (Ctl.) or HMGB1-depleted LCM. Shown are the representative results of two independent experiments and the data are expressed as means ± SEM. **p < 0.01 by student’s *t* test. **(c)** The proposed working model showing that Bcl6 orchestrates transcriptional, epigenetic and metabolic pathways to drive trained immunity in macrophages for tumor progression.

Supplementary Table 1. (F: Forward, R: reverse)

| Target | Primer name | Sequence 5’→ 3’ |
| --- | --- | --- |
| mouse β-actin | β-actin F  β-actin R | CTCATGAAGATCCTGACCGAG  AGTCTAGAGCAACATAGCACAG |
| mouse inos | mouse inos F  mouse inos R | CGGCTGTCAGAGCCTCGTGGCTTTGG  CCTTCCGAAGTTTCTGGCAGCAGCG |
| mouse il-1β | mouse il-1β F  mouse il-1β R | GAAATGCCACCTTTTGACAGTG  TGGATGCTCTCATCAGGACAG |
| mouse il-10 | mouse il-10 F  mouse il-10 R | CTTACTGACTGGCATGAGGATCA  GCAGCTCTAGGAGCATGTG G |
| mouse arg1 | mouse arg1 F  mouse arg1 R | TGAACACGGCAGTGGCTTTA  GCATTCACAGTCACTTAGGTGGTTTA |
| mouse nanog | mouse nanog F  mouse nanog R | ACCTCAGCCTCCAGCAGAT  ACCTCAGCCTCCAGCAGAT |
| mouse notch1 | mouse notch1 F  mouse notch1 R | GCTCCGAGGAGATCAACGAG  TTGACATCACCCTCACACCG |
| mouse sox2 | mouse sox2 F  mouse sox2 R | GCGCGGAGTGGAAACTTTTG  GCGCGGAGTGGAAACTTTTG |
| mouse c-myc | mouse c-myc F  mouse c-myc R | CAGAGGAGGAACAACGAGCTGAAGCGC  TTATG CACCAGAGTTTCGAAGCTGTTCG |
| mouse klf2 | mouse klf2 F  mouse klf2 R | ACCAAGAGCTCGCACCTAAA  GTGGCACTGAAAG GGTCTGT |
| mouse klf41 | mouse klf41 F  mouse klf4 R | ATGGTCAAGTTCCCAGCAAG  GGGCATGTTCAAGTTGGATT |
| mouse hif-1α | mouse hif-1α F  mouse hif-1α R | ACAAGTCACCACAGGACAG  AGGGAGAAA ATCAAGTCG |
| Mouse hk2 | mouse hk2 F  mouse hk2 R | GATTTCACCAAGCGTGGACT  CCACACCCACTGTCATTTG |
| mouse cyclin A1 | mouse cyclin A1 F  mouse cyclin A1 R | ATTGTGCCTTGCCTGAGTGA  GTGCAGGTACTTCGAAGCCT |
| mouse cyclin A2 | mouse cyclin A2 F  mouse cyclin A2 R | CTCGCTGCATCAGGAAGACC  CCCGTCGAGTCTTGAGCTTC |
| mouse cyclin B1 | mouse cyclin B1 F  mouse cyclin B1R | ATGATGGGGCTGACCCAAAC  TTCCAGTCACTTCACGACCC |
| mouse cyclin B2 | mouse cyclin B2 F  mouse cyclin B2 R | GCTAGCTCCCAAGGATCGTC  CTGCAGAGCTGAGGGTTCTC |
| mouse cyclin E1 | mouse cyclin E1 F  mouse cyclin E1R | AAGCGAGGATAGCAGTCAGC  ATTCAAGACGGGAAGTGGGG |
| mouse cyclin E2 | mouse cyclin E2 F  mouse cyclin E2 R | GGAGGAATCAGCCCTTGCAT  ATCTGGCAGAGGTGAGGGAT |
| mouse cdk1 | mouse cdk1 F  mouse cdk1 R | ACGGCGACTCAGAGATTGAC  GGCTTCCACTTGGGAAAGGT |
| mouse cdk2 | mouse cdk2 F  mouse cdk2 R | GTGGTACCGAGCACCTGAAA  CGGGTCACCATTTCAGCAAA |
| mouse cdk4 | mouse cdk4 F  mouse cdk4 R | GACGGTGTACAAAGCCCGA  CAAGGCCACCTCACGAACT |
| mouse cdk6 | mouse cdk6 F  mouse cdk6 R | GTGTCACGGACGGACAGAGA  CGGTTTCAGATCACGATGCAC |
| mouse bcl2 | mouse bcl2 F  mouse bcl2 R | TCTTTGAGTTCGGTGGGGTC  AGTTCCACAAAGGCATCCCAG |
| mouse bak1 | mouse bak1 F  mousebak1 R | GGTGACCTGCTTTTTGGCTG  TTACGGTCAGGATGGGGTCT |
| mouse atp5g2 | mouse atp5g2 F  mouse atp5g2 R | ATGTACGCCTGCTCCAAGTT  CTGTGGTCGCTTCAACTCCA |
| mouse ndufb7 | mouse ndufb7 F  mouse ndufb7 R | GACCCCGAGAAGATACCCAG  GCACAGTAGTCACGTTGCTG |
| mouse sdhb | mouse sdhb F  mouse sdhb R | CAGAGTCGGCCTGCAGTTT  ATCCAACACCATAGGTCCGC |
| mouse sdhd | mouse sdhd F  mouse sdhd R | CTGGTTCCAAGGCTGCATCT  AGCCAGAGAGTAGTCCACCA |
| mouse cox6a1 | mouse cox6a1 F  mouse cox6a1 R | CAACGTGTTCCTCAAGTCGC  CTTCATAGCCGGTCGGAAGT |
| CHIP klf4 | CHIP klf4 F  CHIP klf4 R | CCTTAGCGCGCCTAGTCCTC  CCCAGTCAGTCTGTCAAAGGC |
| CHIP c-myc1 | CHIP c-myc1 F  CHIP c-myc1 R | AG ATGAGAATGGAATCCCGGGTG  GGTGAGCATCCTTAAGAACA |
| CHIP c-myc2 | CHIP c-myc2 F  CHIP c-myc2 R | AGCCGGGTAGAGCGCA  GGTTCGGACTTCCACCCG |
| CHIP c/ebpβ1 | CHIP c/ebpβ1F  CHIP c/ebpβ1 R | GGTTCGGACTTCCACCCG  TCCTGACCTGAAAGCCCATC |
| CHIP c/ebpβ2 | CHIP c/ebpβ2 F  CHIP c/ebpβ2 R | GAGCAGGGTTGAGGGAACAG  TAGGTCGCCATGCACGC |
| CHIP nr4a1-1 | CHIP nr4a1-1 F  CHIP nr4a1-1 R | TGTGAGCAGGCAGATCAGTG  GGAGAAGACACTTTACAAAGGGGA |
| CHIP nr4a1-2 | CHIP nr4a1-2 F  CHIP nr4a1-2 R | ATGTGAGCAGGCAGATCAGTG  TTCCTCGGGAGAAGACACTTT |
| CHIP sox2 | CHIP sox2 F  CHIP sox2 R | CTAGTTGGACAGTCGCCCTG  CATAAGGGTGGATGGGGCG |
| CHIP oct4 | CHIP oct 4 F  CHIP oct 4 R | TGACGAGGATGAACACCGGA  TGGAGATACCCTGCTTCCCTT |
| CHIP tet 1 | CHIP tet1 F  CHIP tet1 R | GACTCTTGGCAGTGCTTGGT  CAGCTTGCCTTGTTCACCTG |
| CHIP tet 2 | CHIP tet2 F  CHIP tet2 R | AAATGCTGTGATTGCAGGTCT  ACCTCTTCTGTGGGATCGGT |
| CHIP tet 3 | CHIP tet3 F  CHIP tet3 R | GCAACCGATCCCACAGAAGA  GAGAGCGCTCAGTTTTCCCT |
| MSP- klf 2 | MSP-klf2 F  MSP- klf 2 R | GAAAGTTTCGTTATTCGTGTTTTTC  CGAACTCAACCTAAATTTAACCG |
| UMSP- klf 2 | UMSP- klf 2 F  UMSP- klf 2 R | GGAAAGTTTTGTTATTTGTGTTTTTT  CCAAACTCAACCTAAATTTAACCAC |
| MSP-oct4 | MSP- oct4 F  MSP- oct4 R | GTAGTGTTTGAGGTGTAGATTTGATC  AAAAAAACTCCTACTACAACAATCG |
| UMSP-oct4 | UMSP-oct4 F  UMSP-oct4 R | GTGTTTGAGGTGTAGATTTGATTGA  AAAAACTCCTACTACAACAATCACT |
| MSP-nr4a1 | MSP-nr4a1 F  MSP-nr4a1 R | GTATGGGCGATTTTGTAGGTTC  AATAACCTAAAATACTAACTCTCGAA |
| UMSP-nr4a1 | UMSP-nr4a1 F  UMSP-nr4a1 R | GTATGGGTGATTTTGTAGGTTTGA  TCACTAAATAACCTAAAATACTAACTCTCA |
